# Catalytic nanosponges of acidic aluminosilicates for plastic degradation and CO_2_ to fuel conversion

**DOI:** 10.1038/s41467-020-17711-6

**Published:** 2020-07-31

**Authors:** Ayan Maity, Sachin Chaudhari, Jeremy J. Titman, Vivek Polshettiwar

**Affiliations:** 1 Department of Chemical Sciences, Tata Institute of Fundamental Research (TIFR), Mumbai, India; 2 School of Chemistry, University Park, University of Nottingham, Nottingham, NG7 2RD UK

**Keywords:** Heterogeneous catalysis, Chemical engineering, Porous materials

## Abstract

The synthesis of solid acids with strong zeolite-like acidity and textural properties like amorphous aluminosilicates (ASAs) is still a challenge. In this work, we report the synthesis of amorphous “acidic aluminosilicates (AAS)”, which possesses Brønsted acidic sites like in zeolites and textural properties like ASAs. AAS catalyzes different reactions (styrene oxide ring-opening, vesidryl synthesis, Friedel−Crafts alkylation, jasminaldehyde synthesis, m-xylene isomerization, and cumene cracking) with better performance than state-of-the-art zeolites and amorphous aluminosilicates. Notably, AAS efficiently converts a range of waste plastics to hydrocarbons at significantly lower temperatures. A Cu-Zn-Al/AAS hybrid shows excellent performance for CO_2_ to fuel conversion with 79% selectivity for dimethyl ether. Conventional and DNP-enhanced solid-state NMR provides a molecular-level understanding of the distinctive Brønsted acidic sites of these materials. Due to their unique combination of strong acidity and accessibility, AAS will be a potential alternative to zeolites.

## Abstract (web summary)

The synthesis of solid acids with strong zeolite-like acidity and textural properties like amorphous aluminosilicates (ASAs) is still a challenge. Here the authors synthesized “acidic amorphous aluminosilicates”, with Brønsted acidic sites like in zeolites and textural properties like ASAs for catalyzing various reactions.

## Introduction

Solid acids are among the most essential heterogeneous catalysts due to their tunable acidity, recyclability, and stability, which have the potential to replace environmentally harmful liquid acids^1–4^. The efficiency of these materials as catalysts depends on their tunable acidity and high surface area for better mass diffusion. Although crystalline zeolites are strongly acidic, they are limited by their inherent microporosity, which introduces diffusion constraints, reducing their performance, particularly in reactions where large molecules are involved^5–12^. Attempts have been made to resolve these issues by synthesizing mesoporous zeolites, but they faced problems of phase separation and stability^9,10^. On the other hand, mesoporous amorphous aluminosilicates (ASAs)^1^ suffered from weak acidity and moderate stability^13–15^.

Acidity in amorphous aluminosilicates and crystalline zeolites results from different types of acidic sites. Zeolites exhibit acidity because of the presence of bridging silanols, where four-coordinated Al(IV) is connected to the silanol oxygen (O) making it negatively charged, and the proton attached to the silanol acts as an acidic site (Supplementary Fig. 1a)^16–18^. For ASAs, the source of the acidity is due to the presence of a pseudo-bridging silanol between the Si and Al (IV) or Al (V) (Supplementary Fig. 1b), which are weaker acidic sites compared to those in zeolites^19–22^. This can be rationalized by the silanol O to Al bond distance, which is much shorter (1.8–2 Å)^22^ in the case of zeolites compared to ASAs (2.94–4.43 Å)^22^. Thus, synthesizing ASAs with zeolite-like bridging silanols can produce materials with strong acidity and accessible surface area. The existence of such a material is still under debate^19,21–23^.

In this work, we synthesize nanosponges of solid acids, named “acidic aluminosilicates” (AAS), which contains strong Brønsted acid sites like zeolites and has high mesoporosity like amorphous aluminosilicates. They have a high surface area of up to 588 m^2^ g^−1^ with a pore volume of 1.5 cm^3^ g^−1^. This unique combination of acidity and accessibility is able to catalyze various challenging reactions more effectively than mesoporous zeolites and ASAs, for the synthesis of large organic molecules, xylene isomerization, plastic degradation, and CO_2_ to dimethyl ether (DME) conversion.

## Results

### Synthesis of AAS

We aim to achieve an efficient hetero-condensation between tetraethylorthosilicate (TEOS) and the Al precursor by choosing suitable reactants with comparable hydrolysis rates^24–27^, and avoid homo-condensation and the formation of two distinct phases. To realize this, we have studied the effect of two different Al precursors having different reactivity, aluminum acetylacetonate (Al-AC) and aluminum isopropoxide (Al-IP)^24^, on their condensation with Si precursor TEOS. Five different materials are synthesized with varying the Si/Al ratio to achieve tunable acidity.

Tuning the acidity of AAS can be achieved by decreasing the Si/Al ratio, to form more number of Brønsted acidic sites (BASs). In addition, by decreasing the particle size as well as by making the material porous, these BASs can be made more accessible. We combined these two approaches in the multistep synthetic protocol, which involves condensation of TEOS and the Al precursor in a bicontinuous microemulsion template made up of lamellar phases of cetyltrimethylammonium bromide (CTAB), and 1-pentanol (Supplementary Fig. 2). Previous work from our group showed that this soft template approach can lead to dendritic morphology of nanosilica with a large pore size distribution (3–25 nm) and high accessible surface area^28–36^. The synthesized materials are named according to the Al precursor used and the Si/Al ratio measured from energy-dispersive X-ray spectroscopic studies. Thus, AC-1.9, 9, and 1.3 means AAS materials synthesized using Al-AC with 1.9, 9, and 1.3 Si/Al ratios, respectively, with CTAB as surfactant and 1-pentanol as cosurfactant. In AC*-1.9 no cosurfactant is used. In IP-0.3, Al-IP is used maintaining the starting precursors molar ratio similar to AC*-1.9.

**Fig. 1:**
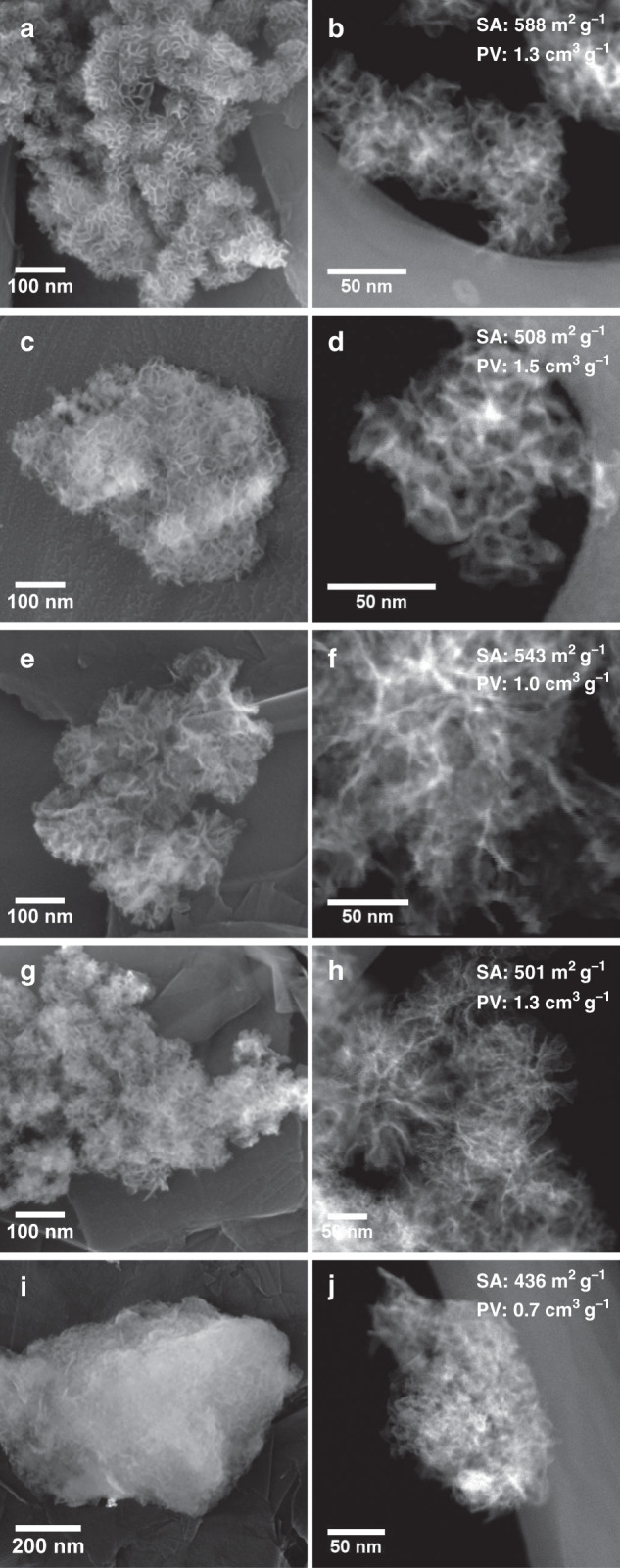
Electron microscope imaging. Scanning electron microscope (first column), HAADF transmission electron microscope images (second column) of the synthesized AAS, and BET surface area (SA) and BJH adsorption pore volume (PV) of **a**, **b** AC^*^-1.9, **c**, **d** AC-1.9, **e**, **f** AC-9, **g**, **h** AC-1.3, and **i**, **j** IP-0.3.

The scanning and transmission electron microscopy images (Fig. 1, Supplementary Fig. 3), indicate the formation of nanoparticles with a porous sponge-like morphology. N_2_ sorption analysis shows AAS exhibit varied textural properties, with surface areas ranging from 436 to 588 m^2^ g^−1^, and pore volumes from 0.7 to 1.5 cm^3^ g^−1^ (Fig. 1, Supplementary Fig. 4). AC*-1.9 has a more accessible and open structure than IP-0.3, with a broad pore size distribution extending up to 25 nm (Supplementary Fig. 4). This indicates the role of Al precursors (AC vs IP) in producing the morphology and textural properties of AAS. Al contents measured using energy-dispersive X-ray spectroscopy (EDX) analysis are in accord with the stability constants of the respective Al precursors^24^. AAS AC*-1.9 contains 15 wt.% Al, while IP-0.3 contains 34 wt.% Al (Supplementary Table 1). This is due to a lower hydrolysis rate for AC compared to TEOS, which leads to a higher Si content. In the case of AAS IP-0.3, the reverse trend is observed due to the higher hydrolysis rate of IP compared to TEOS.

Several other materials are synthesized by varying the Si/Al ratio using the AC precursor (Fig. 1, Supplementary Figs. 3 and 4). Notably, AC*−1.9 and AC-1.9 have a similar Si/Al ratio of 1.9, but differences in their textural properties (pore size distribution), indicating the critical role of cosurfactant on morphology. Supplementary Figure 4 shows that synthesized AAS with different Si/Al ratios ranging from 9 to 1.3 (Supplementary Table 1) have similar nanosponge morphologies (Fig. 1, Supplementary Fig. 3) and pore size distribution. EDX mapping (Supplementary Figs. 5 and 6) indicates a homogenous Al distribution across the nanosponges, while the PXRD and SAED show their amorphous nature (Supplementary Figs. 6 and 7). A few crystalline alumina domains were observed for IP-0.3 only (Supplementary Figs. 6 and 7), but the concentration is too low to observe in PXRD (Supplementary Fig. 7). For comparative study, several well-known solid acids^10,25^ (Supplementary Fig. 8) are also synthesized.

### Acidity of AAS using catalysis as a probe

**Fig. 2:**
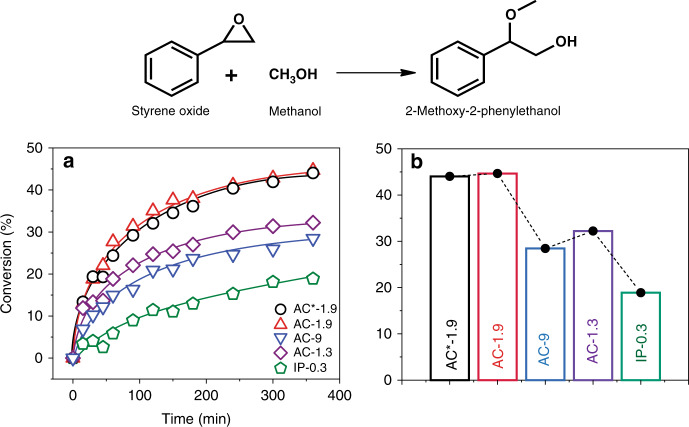
AAS catalyzed styrene oxide ring-opening. **a** Reaction kinetics of AAS catalyzed styrene oxide ring-opening by methanol, **b** total conversion of styrene oxide to 2-methoxy-2-phenylethanol in 6 h of reaction time. Error (s.d.) in the conversion is within ±10%.

To evaluate the overall acidity, ring-opening of styrene oxide, a small molecule, is intentionally chosen so that the role of diffusion in catalytic conversion can be neglected and the strength of the acidic sites can be studied (Fig. 2)^37^. The catalytic performance of AC*-1.9 and AC-1.9 is very similar, and these materials show the highest conversion among all the synthesized AAS (Fig. 2). Notably, with increased Al content, catalytic activity does not increase, with AC-1.9 exhibiting high catalytic conversion and kinetics, as compared to AC-1.3 and AC-9. IP-0.3 is the least catalytically active, indicating the presence of weak acidic sites compared to the other AAS.

Although the above study provided information about the overall acidity of AAS, the styrene oxide ring-opening is catalyzed even by weak acids, and hence this probe reaction is not able to distinguish between the weakly acidic pseudo-bridging and strongly acidic-bridging silanol sites. To study this aspect, we choose the synthesis of vesidryl (2′,4,4′-trimethoxychalcone) that can only be catalyzed by strong sites and requires high mesoporosity because of the larger size of the molecule (Table 1). Both strongly acidic microporous ZSM-5 and weakly acidic mesoporous ASA-2 show poor catalytic activity (Table 1, entry 23 and 25) for this reaction. Even seed-assembled mesoporous materials (SAM)^12^, also known as pseudo-zeolites, catalyze this synthesis with only a moderate conversion (Table 1, entry 30). For better comparison under our experimental conditions, we synthesize MFI-Meso-Zeolite^10^ and then evaluate its catalytic activity. Catalytic performance of Meso-MFI-Zeolite (Table 1, entry 19 and 20) for vesidryl synthesis at 150 °C is similar to AC*-1.9 in 1 h (Table 1, entry 1). This indicates the presence of strong acidic sites like zeolites in AC*-1.9. When the reaction was continued for 24 h, the AC*-1.9 (79% conversion) becomes a superior catalyst over MFI-Zeolite (66% conversion; Table 1, entry 2 and 20) indicating AC*-1.9 not only exhibits stronger acidity, but also has higher accessible sites due to its nanosponge morphology and large pore size distribution. At lower reaction temperature (120 °C), AC*-1.9 exhibits 51% conversion in 4 h, whereas MFI-Zeolite exhibits only 34% conversion (Table 1, entry 11 and 22) further confirming strong acidity and accessibility of AC*-1.9, enhancing the kinetics of reaction even at low temperature. Vesidryl synthesis using commercial ZSM-5 shows negligible conversion (0.9%) in 1 h and 45% conversion in 24 h (Table 1, entry 23 and 24). The significant difference in the catalytic performance at 1 h reaction is probably because, at a lower reaction time point, the reaction is predominantly dominated by acidity, mesoporosity, and accessibility of the catalyst. At a longer time point, a good but slow conversion can be due to the presence of surface acidic sites. Conventional amorphous aluminosilicates also show poor catalytic activity due to their weak acidic sites (Table 1, entry 25–28). All these results indicate the two main aspects of AAS materials, the existence of strong acid sites like zeolites, and superior reaction kinetics because of improved accessibility.

**Table 1:** Vesidryl synthesis catalyzed by AAS and comparison with conventional solid acids.


Entry no.	Catalyst	Temperature (°C)	Time (h)	Yield (%)
Reaction temperature: 150 °C				
1	AC*-1.9	150	1	62
2	AC*-1.9	150	24	79
3	AC*-1.9 (no activation)	150	1	41
4	AC-1.9	150	1	46
5	AC-9	150	1	45
6	AC-1.3	150	1	46
7	IP-0.3	150	1	5
8	IP-0.3	150	24	53
9	IP-2.8	150	1	28
10	IP-2.8 (no activation)	150	1	16
Reaction temperature: 120 °C				
11	AC*-1.9	120	4	51
12	AC*-1.9	120	8	60
13	AC*-1.9 (no activation)	120	4	36
14	AC*-1.9 (no activation)	120	8	48
15	AC-1.9	120	4	40
16	AC-9	120	4	38
17	AC-1.3	120	4	26
18	IP-0.3	120	4	5
Comparison with reported solid acids				
19	Meso-MFI-Zeolite	150	1	58
20	Meso-MFI-Zeolite	150	24	66
21	Meso-MFI-Zeolite (no activation)	150	1	24
22	Meso-MFI-Zeolite	120	4	34
23	ZSM-5	150	1	0.9
24	ZSM-5	150	24	45
25	ASA-2	150	1	28
26	ASA (5/95, cogel)	150	1	20
27	SA (commercial)	150	1	4
28	ASA-2 (1073)	150	1	23
29	Al-MCM-41^10^	150	24	10
30	SAM^10^	150	24	35

Error in the yield is ±5%.

Interestingly all AAS synthesized using the AC precursor shows good conversion (45–62%) in just 1 h, while AAS synthesized using the IP precursor shows poor conversion (Table 1, entry 7 and 9). These results confirm the role of the Al precursor in dictating the nature and strength of the acid sites. The variation in the catalytic activity among AAS synthesized using the AC precursor but with different Si/Al ratio, indicates the presence of different acid sites. To further resolve the acid sites in the AAS, we perform the synthesis of vesidryl at a lower temperature of 120 °C (instead of 150 °C) to reduce the reaction rate. Under these conditions, AC*-1.9 achieve a 51% conversion in only 4 h, which increases to 60% in 8 h (Table 1, entry 11 and 12). At the lower reaction temperature of 120 °C, the catalytic activity of the AAS synthesized using AC is distinguishable, with AC-9 showing a 40% conversion in 4 h, whereas AC-1.3 shows only 26% conversion (Table 1, entry 15–17).

For conventional solid acid catalysts, activation (heating under vacuum) is required to remove adsorbed moisture^10^, whereas, AAS shows similar activity, with better kinetics even without any activation. The synthesized MFI-Meso-Zeolite exhibits only 24% conversion without activation (Table 1, entry 21), while AC*-1.9 shows 41% conversion (Table 1, entry 3). Due to open nanosponge morphology, the reactant molecules efficiently displace water molecules during the reaction, without the requirement of pre-activation of the catalyst.

**Fig. 3:**
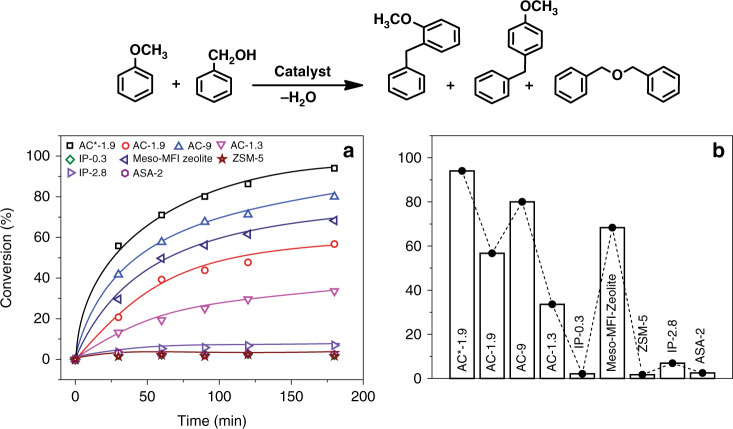
AAS catalyzed Friedel–Crafts alkylation. Solid acid-catalyzed Friedel–Crafts alkylation of anisole by benzyl alcohol at 120 °C, **a** reaction kinetics, **b** total conversion of benzyl alcohol in 3 h reaction time. Error in the conversion is within ±10 %.

To further confirm the presence of strong acidity of AAS, Friedel−Crafts alkylation of anisole by benzyl alcohol, which also requires a strong acidic site and mesoporosity^38^ is carried out (Fig. 3). The best known zeolitized mesoporous aluminosilicates achieved ~50% conversions in 1 h under microwave irradiation at 160 °C using 100 mg of catalyst^38^. Notably, all the five AAS AC series catalysts show 100% conversion in only 15 min using 25 mg of catalyst (Supplementary Table 2). For better comparison, we reduce the catalyst amount and reaction temperature to 10 mg and 120 °C, respectively (Fig. 3, Supplementary Table 3). Results indicates that only AAS AC series and MFI-Meso-Zeolite can catalyze this reaction due to its stronger acidity and mesoporosity, whereas microporous ZSM-5 exhibits negligible conversion (Fig. 3, Supplementary Table 3). Similarly, the other catalysts IP-0.3, IP-2.8, and ASA-2 shows negligible conversion toward this reaction due to the presence of only weakly acidic pseudo-bridging silanol sites. AC*-1.9 shows ~94% conversion, whereas the MFI-Meso-Zeolite exhibits ~70% conversion under the same experimental condition. Although the MFI-Meso-Zeolite has a higher surface area of 812 m^2^ g^−1^ compared to 588 m^2^ g^−1^ of AC*-1.9 (Supplementary Figs. 4 and 8), the significant difference in catalysis is due to the high pore volume (1.3 cm^3^ g^−1^) of AC*-1.9 compare to MFI-Meso-Zeolite (0.6 cm^3^ g^−1^), as well as the wide pore size distribution of AC*-1.9 (Supplementary Figs. 4 and 8). The variation in the catalytic activity among the AC series (Fig. 3) is observed due to the difference in their textural properties and the number of BASs. This catalysis is carried out with no pre-activation process at a high temperature, so there can not be Lewis acidic sites, and hence catalysis is due to BASs only. We have then also compared our material with ASA-2, ASA-2 (1073) that contains a similar amount of the aluminum as in AC*-1.9, and they shows negligible activity for Friedel–Crafts alkylation (Fig. 3, Supplementary Fig. 9). Thus, if Lewis acidic sites are responsible, then these materials should also exhibit good catalytic activity, which is not the case. We also carry out this reaction at 90 °C (to avoid any in situ Lewis site formation) and results further confirm that this catalysis is due to BASs (Supplementary Fig. 9). AC*-1.9 is also compared with well-known amorphous aluminosilicates ASA (5/95 cogel)^25^ and at 90 °C, while 50% conversion is obtained using AC*-1.9 in 24 h, ASA (5/95, cogel) exhibits only 15% of conversion (Supplementary Fig. 9). Thus, combining the results of the vesidryl synthesis and Friedel–Crafts alkylation reaction, it is evident that the AAS AC series possess active sites similar to that of zeolites, with better accessibility.

**Fig. 4:**
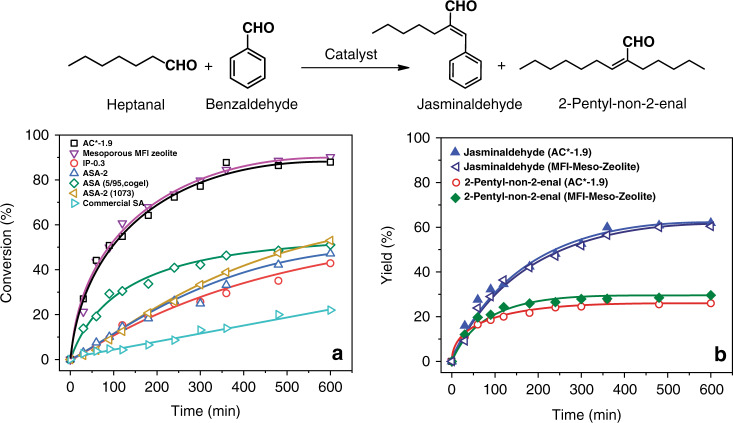
AAS catalyzed jasminaldehyde synthesis. Jasminaldehyde synthesis using solid acids, **a** conversion with respect to heptanal, **b** product yields of best two catalysts, at 125 °C after catalysts activation. Error in the conversion is within ±5% and yield is ±5%.

For further confirmation of the above findings, we carry out jasminaldehyde synthesis (Fig. 4), which also requires strong acid sites like vesidryl synthesis^10^. Even with the jasminaldehyde molecule, AAS AC*-1.9 shows 88% conversion in 6 h, comparable to synthesized MFI-Meso-Zeolite (Fig. 4). A similar activity is also observed without activation (Supplementary Fig. 10b) and even at the lower reaction temperature of 100 °C (instead of 125 °C; Supplementary Fig. 10c). These results suggest that the difference in the reactivity is specific to the type of the reaction as different reaction probes different characteristics of the catalysts, and the overall kinetics and conversion is a complex interplay of several factors, including catalyst textural properties, nature of the active sites and diffusion of the reactant molecules to the active sites.

**Fig. 5:**
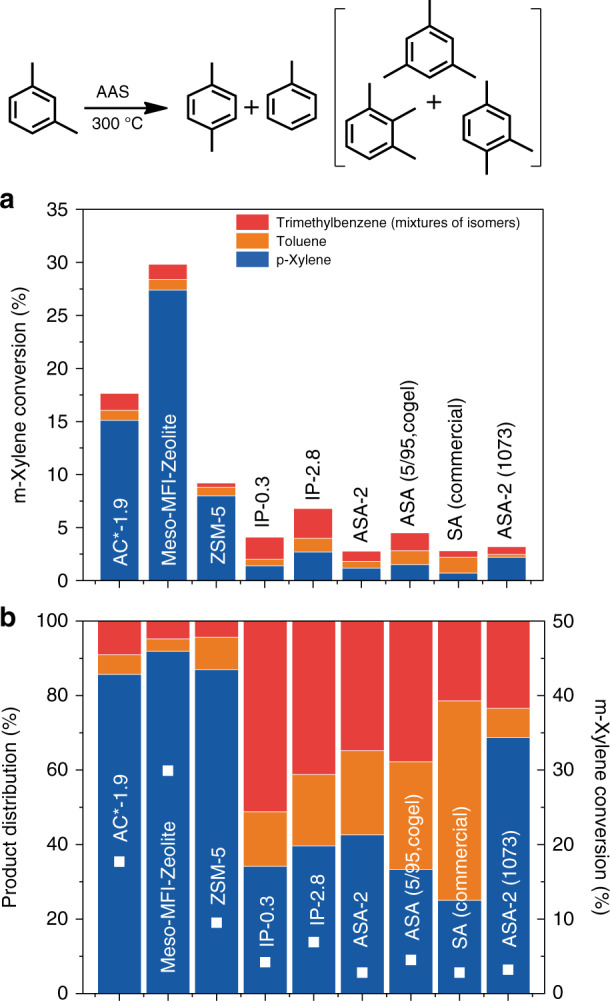
AAS catalyzed m-xylene isomerization. m-Xylene isomerization reaction by solid acids, **a** m-xylene conversion and product yield, and **b** m-xylene conversion (white square) and product distribution. Error in the conversion is within ±10%.

Additional confirmation of the existence of strong BASs in AAS is achieved by conducting m-xylene isomerization as a model reaction (Fig. 5), which was previously catalyzed successfully by zeolites^39–41^. AC*-1.9 converts 18% m-xylene to p-xylene, toluene and trimethylbenzene isomers at 300 °C, while MFI-Meso-Zeolite shows ~29% conversion and commercial ZSM-5 shows 9% conversion. Since m-xylene is a small molecule, diffusion constraints are less compared to other reactions and hence the MFI-Meso-Zeolite showed better performance than AC*-1.9. All the other synthesized materials show much lower conversion than AC*-1.9, which confirms the existence of strong zeolite type acidic sites only in AC series. Further, m-xylene isomerization reaction that selectively probes the BASs is also used to quantify the amount of these sites by catalyst poisoning method using pyridine as the probe molecule^25,39^. The slope of the plot between m-xylene conversion (%) and amount pyridine added (Supplementary Fig. 11), provides the concentration of the acidic sites, which is found to be 225 µmol g^−1^, much higher in magnitude as compared to amorphous solid acids^25,39^.

Another challenging reaction, cracking of isopropylbenzene (cumene) that was known to be catalyzed by zeolites is also carried out by AC*-1.9 and compared with amorphous aluminosilicates (Supplementary Fig. 12). AC*-1.9 shows much higher conversion (~70%) than the conventional ASAs that shows <20% conversion, further confirming the presence of strong BASs in AC*-1.9. Thus, all the six different catalytic studies (styrene oxide ring-opening, vesidryl synthesis, Friedel–Crafts alkylation, jasminaldehyde synthesis, m-xylene isomerization, and cumene cracking) confirms that the AAS contains stronger acid sites like zeolites, and has better accessibility compared to zeolites. These catalytic studies also indicate the critical role of Si/Al ratio, as well as the type of Al precursor in determining the total acidity of AAS. The slower hydrolysis rate of Al-AC precursor as compared to TEOS not only yielded a material with improved accessibility and high surface area, but also increased the effective hetero-condensation between Al and Si precursor creating BASs (bridging silanol) like in zeolites.

### Molecular insights of acidic sites using solid-state NMR

**Fig. 6:**
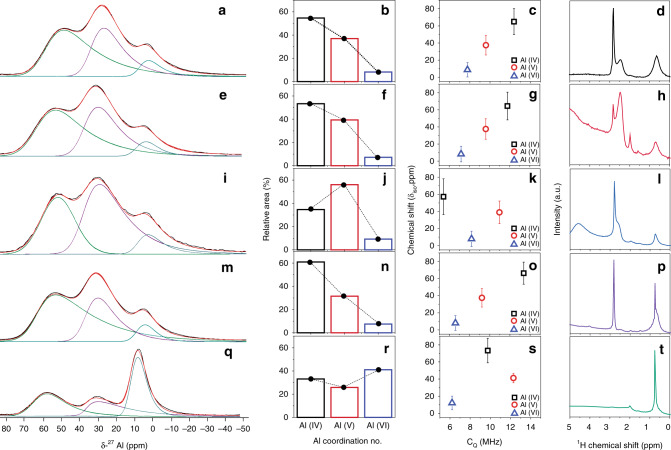
Characterization of AAS by solid-state NMR. ^27^Al direct excitation MAS spectra of AAS samples, **a** AC*-1.9, **e** AC-1.9, **i** AC-9, **m** AC-1.3, and **q** IP-0.3 Decomposition of the spectra was performed using the Gaussian Isotropic Model (Czjzek, *d* = 5) implemented in the DMFit program. Experimental spectra (black trace), total fit (red trace), and deconvolutions of individual sites (lower traces) are shown. The peaks were referenced externally to a 1 M aqueous solution of Al(NO_3_)_3_. Relative peak area of 1D MAS ^27^Al spectra (**b**, **f**, **j**, **n**, **r**), plot of isotropic chemical shift (*δ*_iso_) against quadrupolar coupling constant (*C*_Q_, MHz) (**c**, **g**, **k**, **o**, **s**) for the different sites, single-pulse ^1^H MAS NMR spectra, (**d**, **h**, **l**, **p**, **t**).

The catalytic studies indicate the presence of distinct zeolite-like bridging silanol sites in AAS. To further confirm their existence, one-dimensional (1D) and two-dimensional (2D) solid-state NMR studies are performed, to obtain molecular-level information about the various active sites of AAS. ^27^Al direct excitation low flip angle MAS NMR shows the relative difference in the population between three different types of ^27^Al (Fig. 6a, e, i, m, q). The concentration of tetra-coordinated Al(IV) and penta-coordinated Al(V) sites, which are well-known as the main sources of acidity^19,22^, is higher in the case of AC series, while IP-0.3 had a lower proportion of these sites. The relative population of the Al(VI) nonacidic site is higher (41%) in the case of IP-0.3 as compare to AC series (9%; Fig. 6b, f, j, n, r). However, as IP-0.3 has higher Al contents, the absolute amount of Al(IV) and Al(V) for IP-0.3 is higher than for the AC series (Supplementary Fig. 13), and hence IP-0.3 is expected to be the strongest acid. However, this observation is in disagreement with the catalytic results, where IP-0.3 shows the least activity. This indicates that the nature of acidic sites in IP-0.3 is different from the AC series and the overall acidity of these materials not only depends on the Al coordination number, but also on the presence of proximal silanol and its electronic environment.

To understand the electronic and geometric information of these active sites, the ^27^Al MAS NMR peak shapes and the quadrupolar coupling constants for the different sites are compared. The peak shapes for AAS (Fig. 6) are quite different compared to those observed for amorphous aluminosilicates^22^. With the exception of AC-9, the quadrupolar coupling constant (*C*_Q_) for Al (IV) of the AC series is between 12 and 13 MHz (Fig. 6c, g, k, o), but for conventional amorphous aluminosilicates this is reported as 6 MHz (ref. ^22^). This difference could be due to the combined effect of higher Al content in AAS, as well as the degree of hydroxylation of the Al sites. High *C*_Q_ values are observed for crystalline zeolites (15 MHz)^42–44^, where Al (IV) is connected to the bridging silanol in a crystalline framework. Although AAS is amorphous, the *C*_Q_ value indicates the presence of zeolitic type Al (IV) sites. Sautet et al.^45^ observed the effect of hydroxylation on the *C*_Q_ value in their study of γ-alumina. The hydroxyl density of AAS is calculated by the thermal gravimetric analysis (TGA) of the samples using the Boer equation^46^ (Supplementary Fig. 14, Supplementary Table 4), and found to be within the range of 5.4 to 13 OH nm^−2^.

To understand the nature of Al sites further, proton 1D experiments are performed to study the protons in close proximity with the Al (Fig. 6d, h, l, p, t). It is known that the pseudo-bridging site of amorphous aluminosilicates is responsible for their acidity^19,22,23^. The unique Al (IV) and Al (V) distribution in the AAS materials might perturb the distance between H and Al, leading to a change in the acidity of the proton. The proton 1D spectra shows a clear distinction between the AC series and IP-0.3 (Fig. 6d, h, l, p, t). Proton chemical shifts for conventional ASAs were reported at 0.7 p.p.m. and 1.9 p.p.m. for Al-OH and Si-OH, respectively^17^. Notably, in the AAS AC series, two additional signals are observed at 2.4 and 2.8 p.p.m. (with high intensity), which are absent in the case of IP-0.3 (Fig. 6t).

Interestingly, the peak at 2.8 p.p.m. is much sharper than the peak at 2.4 p.p.m. (Fig. 6d, h, l, p, t), suggesting that the latter arises from a site with more heterogeneity than that at 2.8 p.p.m. Baiker et al.^47^ observed a broadened peak at 2.6 p.p.m. in silica/alumina composite, and attributed it to hydrogen-bonded silanol groups in the vicinity of Al (IV) and Al (V). Mauge et al.^48^ using ^1^H{^27^Al} TRAPDOR experiments showed the existence of a weak shoulder at 2.8 p.p.m. that was attributed to H in close proximity (<5 Å) to Al. In the AAS AC series, the peak at 2.8 p.p.m. has a distinct sharp feature, reconfirming the existence of a zeolitic bridging silanol bond. Now the question arises why such zeolite peaks appear in amorphous AAS? This is due to the efficient hetero-condensation between Si and AC precursor molecules, and the higher population of these sites, which allowed their detection in this NMR study.

**Fig. 7:**
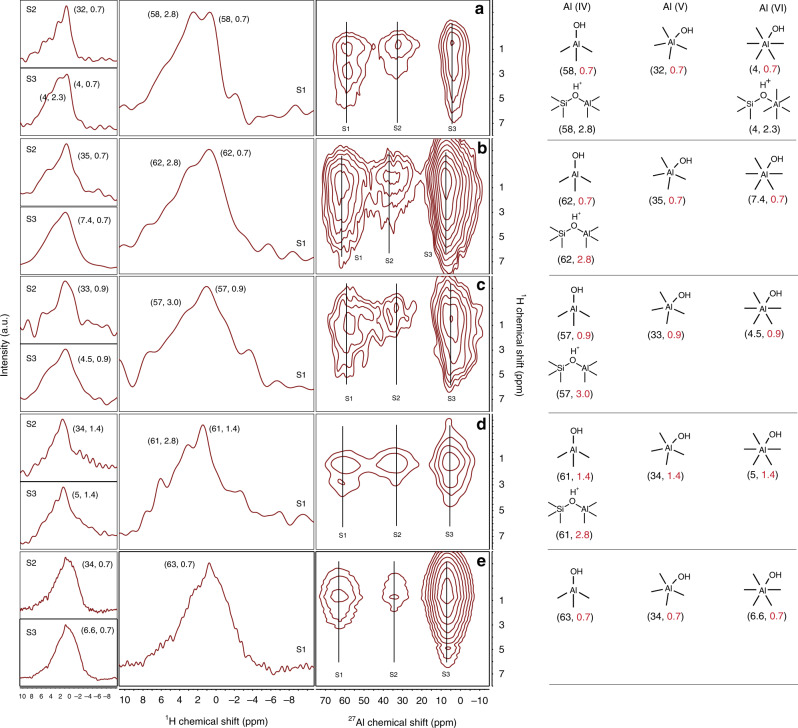
DNP-enhanced ^1^H-^27^Al HETCOR NMR spectra. **a** AC*-1.9, **b** AC-1.9, **c** AC-9, **d** AC-1.3, and **e** IP-0.3; slices of individual contours are shown on the left and proposed structures of Al sites are shown on the right. All spectra were acquired with a 12.0 kHz spinning frequency and a contact time of 0.4 ms.

Furthermore, 2D {^1^H}-^27^Al heteronuclear correlation (HETCOR) experiments can directly probe the connectivity between H and Al, with the appearance of a cross peak indicating close proximity of the two nuclei. However, the sensitivity of conventional NMR was not sufficient to obtain {^1^H}-^27^Al HETCOR spectra. Dynamic nuclear polarization (DNP) surface-enhanced spectroscopy has recently been introduced to characterize such materials with increased sensitivity^22,23^, and so a DNP-enhanced {^1^H}-^27^Al HETCOR experiments are carried out (Fig. 7). It is well-known that with short contact times the HETCOR experiment is selective and that only heteronuclei in close proximity to ^1^H will be excited^17,22,23^, so a short contact time of 400 µs was employed. Cross peaks in the region of (58–63, 0.7–1.4,), (32–35, 0.7–1.4), and (4–7.4, 0.7–1.4) p.p.m. are observed (Fig. 7). These peaks result from surface Al sites and their strong correlation with a directly connected OH group. The strong correlation indicates that the nuclei are in close proximity (<5 Å) like the zeolitic bridging silanol site^17,22,23,47,48^, and the high population of these species can be attributed to the high surface area of these materials. Except for IP-0.3, all the other samples showed a cross peak at (58–63, 2.8–3.0), which is similar to that observed for zeolite Brønsted acid sites^17^. However, the cross peak at (58, 2.8) in AC*-1.9 shows relativity high intensity compared to all the other AAS and notably, AC*-1.9 also exhibits relativity high catalytic activity. This indicates that these unique zeolite acid sites, present in AC*-1.9 in high concentration, contribute toward the stronger acidity and better catalytic activity of this material (Table 1, Figs. 3 and 5).

To further correlate the acidity of the AAS and NMR data, we have conducted an ammonia temperature-programmed desorption (TPD) study (Supplementary Fig. 15). The Gaussian-deconvoluted traces provide quantitative information (Supplementary Table 5) about the acid sites in AAS (Supplementary Fig. 15). In the strong acid region (above 310 °C), the AC series shows two desorption peaks at ~400 °C and 560 °C, whereas in the case of IP-0.3 the desorption peaks are at much lower temperatures of 360 °C and 520 °C, respectively. This indicates that the AC series has a stronger acid site than IP-0.3, as suggested by both catalysis and NMR studies. In addition, H-ZSM-5 is known to show a desorption peak at 400 °C that corresponds to strongly bridging silanol sites^49^, confirming their presence in the AAS. TGA of the samples following exact conditions of TPD experiment (Supplementary Fig. 16e), shows similar weight loss for both the AC*-1.9 and IP-0.3, and hence additional desorption signal intensity in TPD above 310 °C must be due to ammonia desorption. TPD measurements without ammonia also confirm this observation (Supplementary Fig. 16). Pyridine adsorption studies using diffuse reflectance infrared Fourier transform spectroscopy also confirm the presence of BASs^39^. DRIFT spectra of adsorbed pyridine on AC*-1.9 (Supplementary Fig. 17), shows the bands at 1546 cm^−1^ and 1638 cm^−1^ corresponds to pyridinium ions, indicating the binding of pyridine molecule with strong Brønsted sites.

### Application of AAS for plastic degradation

**Fig. 8:**
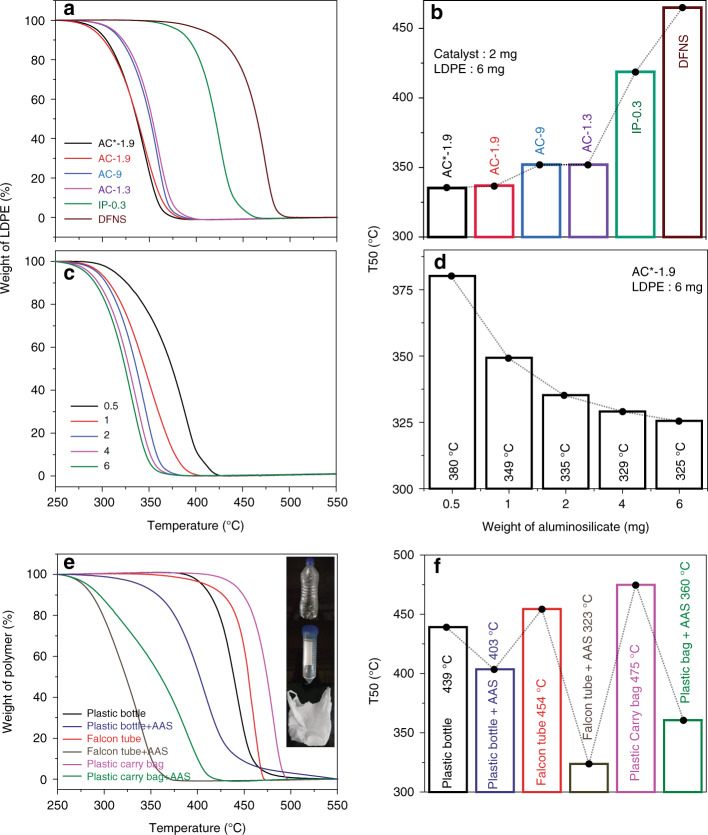
Household and laboratory plastic degradation using AAS. **a** Weight loss curve of LDPE pyrolysis using AAS and DFNS; **b** T50’s of the LDPE pyrolysis using LDPE (6 mg) and AAS AC*-1.9 (2 mg); **c** weight loss curve and **d** T50, of LDPE (6 mg) pyrolysis by varying the catalyst (AC*-1.9) amount to 0.5, 1, 2, 4, and 6 mg; **e** weight loss curve of plastic bottles, falcon tubes, and plastic carrier bags (6 mg each), and **f** T50, using AAS AC*-1.9 (2 mg). Error in the T50 is ± 3 °C.

An excessive amount of plastic waste has become a serious environmental problem. To take on this challenge, we explore the use of AAS to degrade plastic sustainably and have studied the catalytic pyrolysis of low-density polyethylene (LDPE) to hydrocarbons (fuel; Fig. 8). The performance of various AAS for LDPE pyrolysis is evaluated by comparing three different temperatures, T5, T20, and T50, at which 5%, 20%, and 50% of LDPE mass are pyrolyzed to hydrocarbons (Supplementary Fig. 18). These values provided three different time points for the pyrolysis process, with T5 and T20 representing the initial kinetics, where cracking occurs predominantly due to the external surface acid sites. At T50, the degraded polymeric species diffuses into the pores of the AAS for further degradation, and this point provides information about the accessibility of the interior acid sites.

The performance of the AAS for plastic pyrolysis also follows a similar trend that of their catalysis (Fig. 8, Supplementary Fig. 18). In the control experiment, a nonacidic dendritic fibrous nanosilica (DFNS)^28,29^ does not show any catalytic behavior in these degradation experiments, indicating the requirement of acidic sites for plastic degradation process. The lowest T50 value among the AAS is found to be 335 °C for AC*-1.9 (Fig. 8a, b), with fast degradation kinetics (Fig. 8a). This is due to the strong acid sites, as well as the efficient diffusion of melted plastic inside the AAS nanosponges. By optimizing the LDPE to AC*-1.9 ratio, T50 is further reduced to 325 °C (Fig. 8d, Supplementary Fig. 18c).

During a recyclability study with AAS AC*-1.9 for LDPE degradation, an increase in T50 is observed with an increasing number of cycles. In the 15th cycle, the T50 increase from 345 °C to 382 °C (Supplementary Fig. 19). The reason for such an increase is carbon formation on the surface of the catalyst that blocked the active sites. However, after oxygen treatment to burn away the carbon, the T50 returns to a value of 351 °C (Supplementary Fig. 19). Recyclability is studied up to the 27th cycle and the catalyst retains its activity, with a minor deactivation, which can be regenerated by simple oxygen treatment (Supplementary Figs. 19 and 20).

The universality of AAS AC*-1.9 is studied via the degradation of plastic in daily household and laboratory use, such as plastic bottles, centrifuge tubes, and carrier bags (Fig. 8e, f). Notably, the T50 of falcon tubes is reduced to 323 °C, (from 454 °C), carrier bags to 360 °C (from 475 °C), and plastic bottles to 403 °C (form 439 °C). The moderate reduction in the T50 of the plastic bottle is because, while falcon tubes and carrier bags are predominantly made of hydrocarbons, plastic bottles are made from polyethylene terephthalate (PET; Supplementary Table 6). Thus, AAS catalyzed the degradation of polymers containing a hydrocarbon monomer most effectively.

**Fig. 9:**
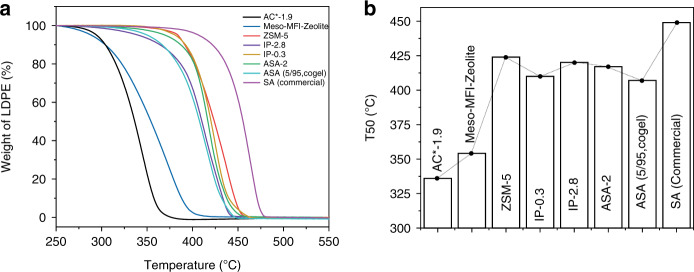
Comparison of solid acids for LDPE pyrolysis. **a** Weight loss curve, **b** T50’s, using various solid acids (2 mg) and LDPE (6 mg). Error in the T50 is ±3 °C.

Notably, AC*-1.9 shows better performance than the well-known zeolite USY-2.6, with T50 equal to 341 °C (ref. ^50^). It also showed superior performance than several other solid acids, as summarized in Supplementary Table 7. The AC*-1.9 is then compared with the other synthesized solid acids. Notably, T50 of AC*-1.9 (~335 °C) was lower then MFI-Meso-Zeolite (~350 °C), as well as of other catalysts (Fig. 9). These results again confirm the stronger acidity of the AAS due to the zeolitic bridging silanols, as well as the accessible acid sites because of the high surface area and a sponge-like morphology.

### Application of AAS for CO_2_ conversion

**Fig. 10:**
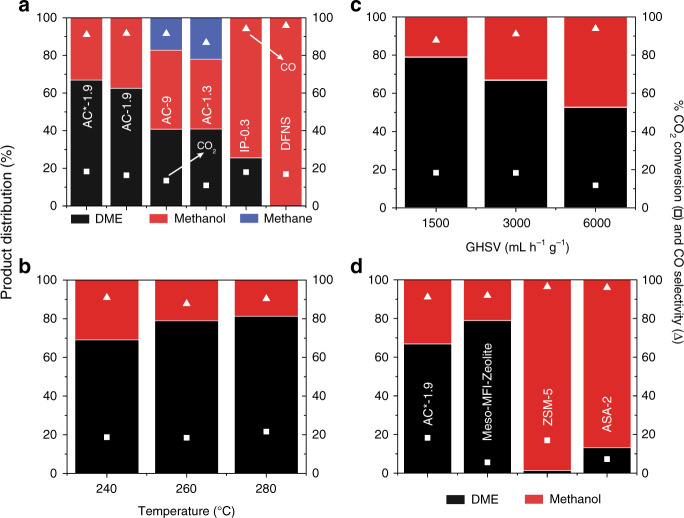
AAS catalyzed CO_2_ to DME conversion. **a** CO_2_ conversion, CO selectivity, and product distribution (DME, methanol, and methane) of various Cu-Zn-Al/AAS hybrid catalysts; **b** effect of GHSV; and **c** Effect of temperature on the product distribution (DME and methanol) of CO_2_ hydrogenation using AC*-1.9 as a catalyst. **d** CO_2_ conversion, CO selectivity, and product distribution (DME and methanol) of various Cu-Zn-Al/aluminosilicate hybrid catalysts. Square and triangle symbol represent CO_2_ conversion and CO selectivity. Error in the CO_2_ conversion is ±10% and in the DME selectivity is ±6%.

Another societal challenge that we are facing is climate change due to excessive atmospheric CO_2_. The use of Cu-Zn-Al/AAS to reduce CO_2_ from the atmosphere and its conversion to DME^51^ is also explored, by preparing hybrid bifunctional catalysts, Cu-Zn-Al/AAS (Supplementary Fig. 21, Supplementary Table 8). The Cu-Zn-Al catalyst converts CO_2_ to methanol, and then the acid sites in AAS convert the methanol to DME. Selectivity for DME formation was known to depend on acidity^51^. Catalysis is conducted using a flow reactor with CO_2_ and a hydrogen ratio of 1:3 and 3000 mL h^−1^ g^−1^ GHSV at 260 °C under 30 bar pressure (Fig. 10a). Various AAS shows CO_2_ conversion between 15% and 19%. As expected, Cu-Zn-Al/DFNS shows no formation of DME due to the absence of acid sites in DFNS. Cu-Zn-Al/AAS shows a variation in selectivity, with the highest DME formation (~68%) achieved in the case of AC*-1.9 and AC-1.9. By optimizing the GHSV to 1500 mL h^−1^ g^−1^, DME selectivity is increased to 79%. This is due to higher residence time, in turn allowing more molecules of methanol to react with AAS to form more DME (Fig. 10b). A decrease in reaction temperature decreases DME production activity, whereas DME production is almost unchanged upon increasing the temperature (Fig. 10c). The catalyst stability is also studied under the stream for 150 h using 1500 mL h^−1^ g^−1^ GHSV at 260 °C, and it is found to be moderately stable with only a 20% reduction in DME selectivity (Supplementary Fig. 22) after 150 h. These results confirm the strong acidity, stability, and accessibility in AAS AC*-1.9, as well as its usefulness for CO_2_ to DME conversion. The best catalyst AC*-1.9 is then compared with the reported solid acids. AC*-1.9 shows better CO_2_ conversion than MFI-Meso-Zeolite, whereas both of them show similar selectivity for DME; while ZSM-5 and ASA-2 show poor DME selectivity (Fig. 10d).

## Discussion

In this work, by using the techniques of bicontinuous microemulsion droplets as a soft template, we synthesized “AAS” with a nanosponge morphology, exhibiting both zeolitic (strong acidity) and amorphous aluminosilicate (mesoporous high surface area) properties. The presence of zeolite-like bridging silanol in AAS was proved by six different catalytic reactions (styrene oxide ring-opening, vesidryl synthesis, Friedel–Crafts alkylation, jasminaldehyde synthesis, m-xylene isomerization, and cumene cracking) that requires strong acidic sites and larger pore sizes. The synergy between strong acidity and accessibility was reflected in the fact that AAS showed better performance than state-of-the-art zeolites and amorphous aluminosilicates. This was also confirmed by detailed solid-state NMR studies and ammonia TPD studies. Thus, based on the catalysis, solid-state NMR and TPD studies, it was clear that the AAS AC series possess strongly acidic zeolite-like bridging silanol sites, even though materials are not crystalline but amorphous. They, therefore, fall into a class of materials at the interface between crystalline zeolite and amorphous aluminosilicates.

AAS was also useful for plastic degradation and converted plastic to hydrocarbons at a temperature, 100 °C less than the temperature in the absence of a catalyst. AAS also played a critical role in CO_2_ to DME conversion using bifunctional Cu-Zn-Al/AAS hybrid catalysts and was able to yield DME with 79% selectivity.

Thus, these unique AAS possesses strong acidity like zeolites and textural properties like aluminosilicates, with nanosponge morphology. Due to their strong acidity and tunable textural properties, they can be used for a range of catalytic reactions, including solution phase and fixed-bed flow processes. Their mesoporosity and nanosponge morphology allowed excellent mass diffusion compared to mesoporous zeolites. Due to the combination of strong acidity and accessible textural properties, AAS may fill the gap between crystalline and amorphous solid acids finding applications in various fields.

## Methods

### Synthesis of acidic aluminosilicates

In a typical synthesis, CTAB (500 mg) and urea (600 mg) were mixed with 5 mL of water for 30 min using magnetic stirring (1400 r.p.m.). A freshly prepared solution of TEOS and Al precursor (as per Table 2) in p-xylene (45 mL) was added dropwise, and stirred for 30 min at room temperature (RT). 1-Pentanol (1.5 mL) was then added dropwise under stirring and the reaction mixture was further stirred for 30 min. It was then refluxed at 120 °C for 12 h and subsequently cooled to RT. The solid product was isolated by centrifuge and was washed four times with ethanol, and three times with water followed by another ethanol washing. Samples were dried overnight at 80 °C. CTAB template was then removed by calcination at 750 °C (ramp 5 °C min^−1^) for 6 h in air.

**Table 2:** Moles of TEOS and Al precursor for the synthesis of AAS with various Si/Al ratios.

Sample name	TEOS (mmol)	Al precursor (mmol)	Cosurfactant (1-pentanol, mL)
AC*-1.9	2.8	2.8	No cosurfactant added
AC-1.9	2.8	2.8	1.5
AC-9	5.3	0.3	1.5
AC-1.3	1.9	3.7	1.5
IP-0.3	2.8	2.8	1.5
IP-2.8	5.4	1	1.5

### AAS catalyzed plastic pyrolysis

LDPE (Alfa Aesar, particle size 500 µm and density 0.92 g cm^−3^), plastic bottle (chemical composition: PET), centrifuge tube (chemical composition: polypropylene), and carrier bag (chemical composition: high-density polyethylene) were used. Pyrolysis studies were performed by TGA (Mettler Toledo), in which the AAS and LDPE or plastic bottle (flakes) or carry bag (small pieces) were mixed in a mortar pestle, and loaded in an alumina crucible. The pyrolysis reaction was carried out in a nitrogen flow of 70 mL min^−1^ from 250 °C to 550 °C (ramp 10 °C min^−1^) temperature in TGA.

### AAS/Cu-Zn-Al catalyzed CO_2_ to dimethyl ether conversion

CO_2_ to DME conversion was performed in a fixed-bed reactor with an inner diameter of 9 mm using 500 mg catalyst. A bifunctional AAS/Cu-Zn-Al catalyst was prepared by physically mixing AAS (200 mg) and Cu-Zn-Al **(**300 mg). The reaction was carried out under 30 bar pressure using gas flows as follows, CO_2_: 5 mL min^−1^, H_2_: 15 mL min^−1^, N_2_: 5 mL min^−1^, and GHSV: 3000 mL h^−1^ g^−1^ at 260 °C. The reactor was connected to an online GC with a heated gas sampling line (Supplementary Fig. 23). The reaction was monitored over time, and the products were identified by GC (Agilent 7890B GC) equipped with a CP7430 column. CO_2_, CO, and N_2_ were analyzed by TCD, and the hydrocarbons by FID. The catalytic performances after 60 min on stream were used for quantification.

## Supplementary information

Supplementary Information

## Data Availability

The data that support the findings of this work are available within the article and its Supplementary Information files.
