# Supplementary material for: Catalytic nanosponges of acidic aluminosilicates for plastic degradation and CO2 to fuel conversion
Source: Nat Commun. 2020 Jul 31;11:3828. doi: 10.1038/s41467-020-17711-6 (PMC7395177; doi:10.1038/s41467-020-17711-6)
Supplement: Supplementary file 1 — Supplementary Information [file 41467_2020_17711_MOESM1_ESM.pdf]

Supplementary Information

Catalytic Nanosponges of Acidic Aluminosilicates for Plastic Degradation and CO_2 to Fuel Conversion

Ayan Maity,[1] Sachin Chaudhari,[2] Jeremy J. Titman,[2] and Vivek Polshettiwar[1]*

[1]Department of Chemical Sciences, Tata Institute of Fundamental Research (TIFR), Mumbai, India

[2]School of Chemistry, University Park, University of Nottingham, Nottingham NG7 2RD, UK

Supplementary Methods

Characterization. All SEM imaging was performed using a Zeiss Ultra microscope at 10kV with a working distance of 3 mm. TEM imaging, EDX was performed with FEI Titan microscope at 300 kV. N_2 sorption studies were performed in a 3-Flex surface analyzer. Before the analysis samples were degassed at 120 °C for 12 h under 0.18 mmHg vacuum followed by in-situ degassing at 120 °C for another 2 h under 0.000119 mmHg vacuum.

AAS Catalyzed Styrene Oxide Ring Opening by Methanol. AAS (15 mg) was taken in a Schlenk flask, degassed at 120 °C for 1h, and subsequently cooled under N_2. Absolute methanol was added, and the flask was brought to at 40 °C, under N_2. Styrene oxide (500 µl) was added to this flask under stirring and the reaction progress was monitored by withdrawing samples at different time points (0, 15, 30, 45, 60, 90, 120, 150, 180, 240, 300 and 360 minutes). All the products were identified by an Agilent 7890B GC-MS.

AAS Catalyzed Synthesis of 2',4,4'-Trimethoxychalcone (Vesidryl). Vesidryl synthesis was carried out at two different temperatures, 120 °C and 150 °C. AAS was taken in a Schlenk flask, degassed at 120 °C for 1h, and subsequently maintained under N_2 at the required reaction temperature. p-Anisaldehyde was dissolved in 2,4-diethoxy acetophenone and then added to the reaction flask. Each of these reactions was conducted for various reaction times, and products were identified and quantified by GC-MS. Catalyst, p-anisaldehyde and 2,4-diethoxy acetophenone amounts used were 50 mg, 7 mmol and 7 mmol for the reaction at 150 °C, and 20 mg, 3.7 mmol and 3.4 mmol for the reaction at 120 °C, respectively.

AAS Catalyzed Friedel−Crafts Alkylation of Anisole by Benzyl Alcohol. The catalysis was performed in two different ways, one is in a microwave reactor and another in an oil bath. The 25 mg AAS mixed with 25 mmol of anisole and 1.68 mmol of benzyl alcohol in a 10 mL glass reactor tube and then irradiated with microwave (200W) for 15 minutes maintaining the reaction temperature at 160°C. In the oil bath condition, 25 mmol of anisole and 1.68 mmol of benzyl alcohol is mixed with 10 mg of catalyst and dipped into a pre-heated oil bath at 120°C and the reaction progress was monitored for 3h under stirring condition.

AAS Catalyzed Synthesis of Jasminaldehyde. AAS (50 mg) was taken in a Schlenk flask and degassed at 120°C for 1h and subsequently maintained under N_2 at 125 °C. Benzaldehyde (15 mmol) and heptanal (3 mmol) were pre-mixed and then added to the reaction flask. Reaction progress at 125 °C was monitored by GC-MS.

m-Xylene Isomerization. m-Xylene isomerization was carried out underflow condition (in HEL Auto-MATE reactor). The catalyst (200 mg) was hated to 300°C (ramping rate 2.5°C min^{-1}) under a nitrogen flow of 5 mL min^{-1}. As soon as the temperature reached 300°C, the N_2 flow was switched to m-xylene vapors (5 mL min^{-1} nitrogen flow bubbled through 15 mL m-xylene) and the products from the gas outlet was monitored by an Agilent 7890B GC-MS system. The catalytic performances after at least 90 min on stream were used for quantification.

Cumene (Isopropylbenzne) Cracking. The reaction was carried out underflow condition (in HEL Auto-MATE reactor), in which 200 mg of aluminosilicate was hated to 300°C (ramping rate 2.5°C min^{-1}) under a nitrogen flow of 5 mL min^{-1}. As soon as the temperature reached 300°C, the gas flow was changed to cumene vapors (5 mL min^{-1} nitrogen flow bubbled through 12 mL cumene), and the products from the reactor outlet were monitored by an Agilent 7890B GC-MS system.

Synthesis of MFI-Meso-Zeolite. Sodium aluminate (500 mg), NaOH (2 g), tetrapropylammonium bromide (7 g) and water (338 mL) were mixed at 600 rpm for 30 minutes. To this transparent solution, a freshly prepared solution of tetraethylorthosilicate (21.4 g) and [3-(trimethoxysilyl)propyl]hexadecyldimethylammonium chloride (2.9 g, 66% methanol solution) was added slowly at RT under vigorous stirring (at 800 rpm) and stirring was continued for another 2h for uniform mixing. The mixture was then hydrothermally treated in an autoclave for 30 h at 150°C. The solid product was isolated via centrifugation and washed 10 times with DI water (50 ml each time) and dried at 80°C overnight in a hot air oven. Then it was calcined in air at 550°C (ramp rate 5°C/min) for 6 h to remove the organic template. The obtained solid was then ion-exchanged by refluxing with 1M of NH_4NO_3 three times and then subsequently washed with DI water then dried and calcined at 550°C for 4h.

Synthesis of ASA-2. Silica gel (100-200 mesh size, 500 mg) was degassed for 2h at 120°C for removal of the adsorbed water and then at RT 20 mL isopropanol was added. To this reaction mixture, aluminum isopropoxide (200 mg) was added and the whole mixture was refluxed for 5h. The solid product was then isolated via centrifugation and washed several times with ethanol and dried at 80°C overnight. Then it was calcined in at 550°C for 5h (ramp rate 2.5°C min^{-1}). Another part of uncalcined ASA-2 was calcined at 800°C for 4h and the sample was designated as ASA-2 (1073).

Synthesis of ASA (5/95, cogel). This synthesis was carried out by following the exact protocol reported in the literature.[26] Sodium silicate solution (Sigma, 26.5% SiO_2) was added to a solution of aluminum

trichoride ($AlCl_3.6H_2O$, 102 mg in 100 mL water) under stirring at 700 rpm. After 10 minutes of stirring, the pH was adjusted to 7 by adding the required amount of glacial acetic acid. The resultant solution was kept under stirring for one hour and then the solid was isolated via centrifugation and washed 10 times with water (200 mL for each step). Then the ion exchange was carried out by refluxing the solid with 0.3M NH_4NO_3 solution seven times. The obtained solid was isolated and then washed again with water once and then dried at 120°C for 8h and then calcined in static air at 650°C for 4h.

ZSM-5. It was commercially purchased from Thermo Fisher Scientific, Product Code: 279571000, CAS 308081-08-5, and Lot: A0391828.

Commercial Silica-Alumina. It was purchased from Micromeritics, Product Code. 004/16821/00 and Lot: A-501-57.

Alumina NP (~50 nm). It was purchased from Allied Hightech, Product Code. 90-187505 and Lot: M013018/E19JW.

Acidity Quantification of AC*-1.9 by Catalyst Poisoning. Five powder samples of AC*-1.9 were treated with varying amounts of pyridine. For this, 200 mg of the AC*-1.9 was first degassed at 100°C for 2h to remove adsorbed moisture and cooled to 30°C under nitrogen flow. It was then treated with varying amounts of a freshly made stock solution of pyridine in dichloromethane (DCM). The mixture was then heated at 50°C under stirring for 30 min and then dried at 80°C in an oil bath to remove the DCM. The obtained dried powder was used for m-xylene isomerization reaction as per the above experimental conditions. m-Xylene conversion (%) was plotted against the amount (μmol/g) pyridine used to treat the AC*-1.9. The amount of the treated pyridine at which the conversion reached to zero was considered as the concentration of the acidic sites.

Synthesis of Cu-Zn-Al Catalyst. $Cu(NO_3)_2.3H_2O$ (36.9 g), $Zn(NO_3)_2.6H_2O$ (22.8 g), $Al(NO_3)_3.9H_2O$ (13 g) were dissolved in 200 mL deionized water and stirred for 30 minutes at 50 °C. To this solution, a previously prepared $Na_2CO_3.10H_2O$ (28.6 g in 50 mL DI water) solution was added dropwise until the pH reached 7, at 50 °C. The reaction mixture was then stirred 12 h at 50 °C. The solid precipitate was isolated by centrifugation and washed five times with water followed by washing using ethanol. Catalysts were dried at 80 °C for 12 h and then calcined at 550 °C for 6 h in air.

Solid-State NMR. Solid-state NMR experiments were performed on 600 (14.1 T) and 850 MHz (20.0 T) Bruker AVANCE II/III spectrometers, equipped with triple resonance HXY and double resonance HX 2.5

mm MAS probes, respectively. The MAS rate was regulated to ±5 Hz and the sample temperature to 300±2 K. Zirconia MAS rotors were used for all the solid-state NMR experiments. All the samples were degassed at 120 °C for 4 h and then rotors were packed in a glove box.

^{1}H spectra were acquired at a Larmor frequency of 600 MHz and a MAS rate of 30 kHz, using a DEPTH pulse sequence to remove background signal. The π/2 pulse length was 2.5 μs. Relaxation times (T_1) were measured with a standard saturation recovery sequence followed by an echo period before the signal acquisition.

^{27}Al spectra were acquired at a Larmor frequency of 221.5 MHz using a pulse length of 1.7 μs corresponding to a flip angle of 30°, allowing for the acquisition of quantitative data. The MAS rate was 30 kHz. The spectra were referenced externally to a 1M aqueous solution of $Al(NO_3)_3$. Relaxation times (T_1) were measured with a standard saturation recovery sequence followed by an echo period before the signal acquisition to ensure quantitative data.

DNP-Enhanced Solid-state NMR. For DNP studies, approximately 65 mg of powdered sample was wetness impregnated with 45 μL of 16 mM TEKPol solution in 1,1,2,2-tetrachloroethane. The wet solid (50 mg) was packed into a 3.2 mm zirconia rotor. DNP-enhanced solid-state NMR experiments were performed on a 600 MHz (14.1 T) Bruker AVANCE III HD spectrometer, equipped with a triple resonance 3.2 mm low-temperature MAS probe. DNP was achieved by irradiating the sample with high-power microwaves (10 W at the probe) at a frequency of 395 GHz, generated by a gyrotron operating continuously with a stability of better than ±1%. The MAS rate was regulated to 12 kHz ± 20 Hz and the sample temperature to 100 K ± 5 Hz. Thin-walled zirconia rotors were used for all DNP experiments.

^{1}H spectra were acquired with 100 kHz rf amplitude excitation, using a rotor-synchronized spin-echo sequence. All experiments were performed with a train of saturation pulses prior to a longitudinal relaxation delay followed by signal excitation. DNP build-up times (T_{DNP}) were measured with a standard saturation recovery sequence followed by an echo period before the signal acquisition under microwave on conditions.

DNP-enhanced $\{^{1}H\}$-^{27}Al HETCOR spectra of samples were recorded with a MAS rate of 12 kHz. The CP contact time was 400 μs, and the relaxation delay was 7.4 s. An SW(f)-TPPM heteronuclear decoupling sequence was applied during t_2 with an RF amplitude of 100 kHz. During t_1 PMLG homonuclear decoupling was applied with an RF amplitude of 100 kHz. A scaling factor of 0.46 was applied after processing to correct the ^{1}H chemical shift scale.

Ammonia Temperature Programmed Desorption (NH_3-TPD) Study. The analysis was performed using a flow-through microreactor system equipped with TCD (BELCAT II). Around 50 mg of the sample was degassed at 120 °C for 1 h under He flow (50 mL min^{-1}) and the temperature was reduced to 100 °C. The activated sample was then exposed to 5% NH_3 in Helium (30 mL min^{-1}) at 100 °C for 30 minutes. Then a fraction of the adsorbed NH_3 was desorbed by passing the He (50 mL min^{-1}) gas for 15 minutes. From the start temperature (100 °C), the reactor was heated to 700 °C with a ramp of 10 °C /min under He flow of 30 mL min^{-1}.

Thermal Gravimetric Analysis of the AAS. The analysis was performed using a Mettler Toledo TGA instrument. The temperature program was kept exactly the same as that of TPD, except the gas used was nitrogen. In a typical analysis, 3-6 mg of sample was taken in an alumina crucible and was degassed at 120°C for 1 h under N_2 flow (50 mL min^{-1}). The temperature was then reduced to 100°C and gas flow was reduced to 30 mL min^{-1}, the system was stabilized for 45 minutes. From 100°C, the chamber was heated up to 700°C at the rate of 10°C min^{-1} under 30 mL min^{-1} N_2 flow.

Pyridine adsorption study using DRIFT. This experiment was performed using a Jasco IR spectrometer equipped with PIKE DRIFT accessories. Approximately 10 mg sample was taken in a crucible and degassed to remove moisture at 450°C under a nitrogen flow of 10 mL min^{-1} for 1h in the DRIFT chamber. Then the temperature was reduced to 50°C and pyridine vapor (using a flow of 10 mL min^{-1} N_2) was passed through the sample chamber for 30 minutes. The weakly adsorbed pyridine was then removed by increasing the temperature to 120°C and 240°Cunder N_2 flow of 20 mL min^{-1} for 1 h. DRIFT spectrum was then recorded at 120°C temperature with a resolution of 4 cm^{-1}.

Supplementary Figures

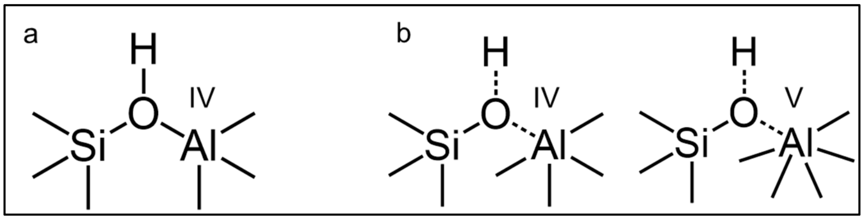

Supplementary Figure 1. Schematic of acidic sites in (a) zeolites and (b) ASAs.

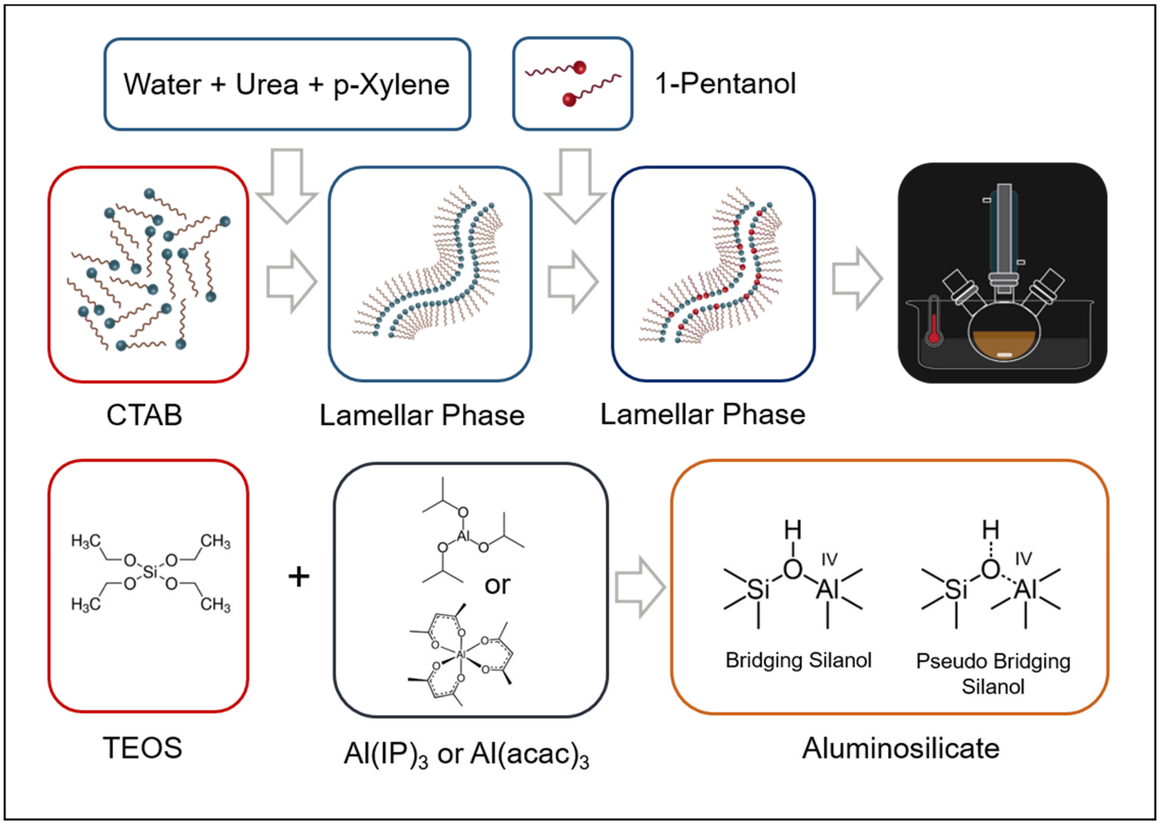

Supplementary Figure 2. Schematic illustration of the acidic amorphous aluminosilicate.

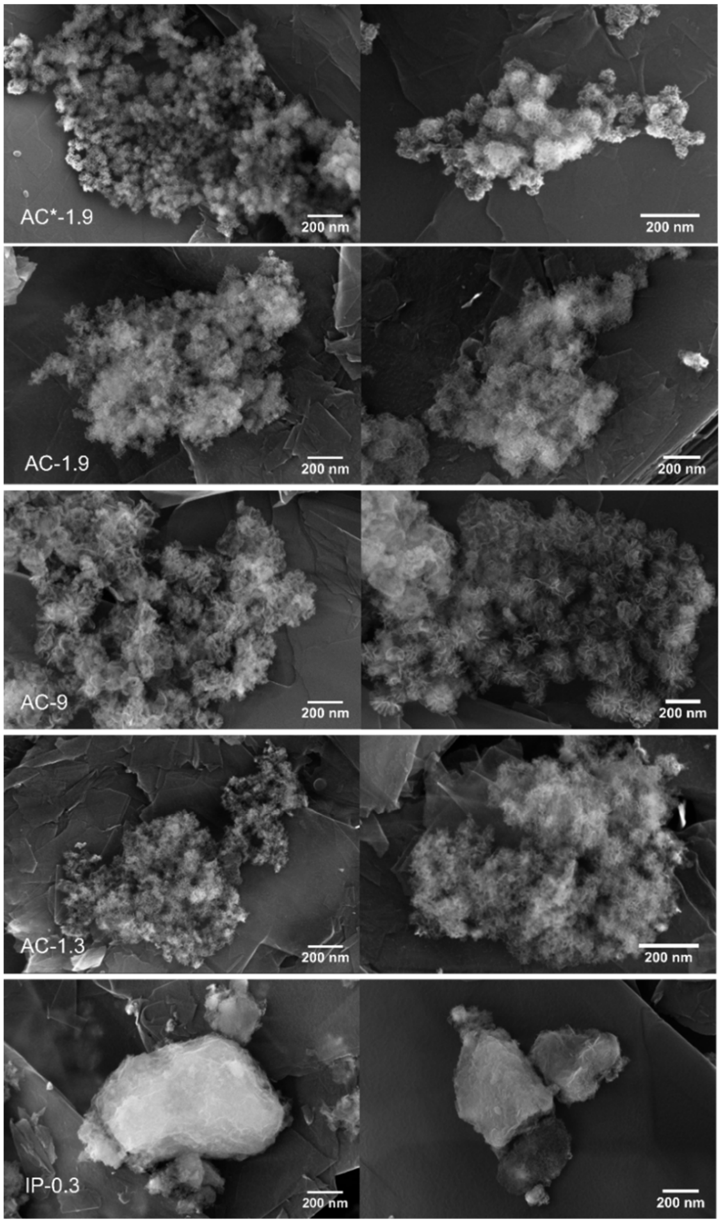

Supplementary Figure 3. Additional SEM images of AC and IP series of AAS.

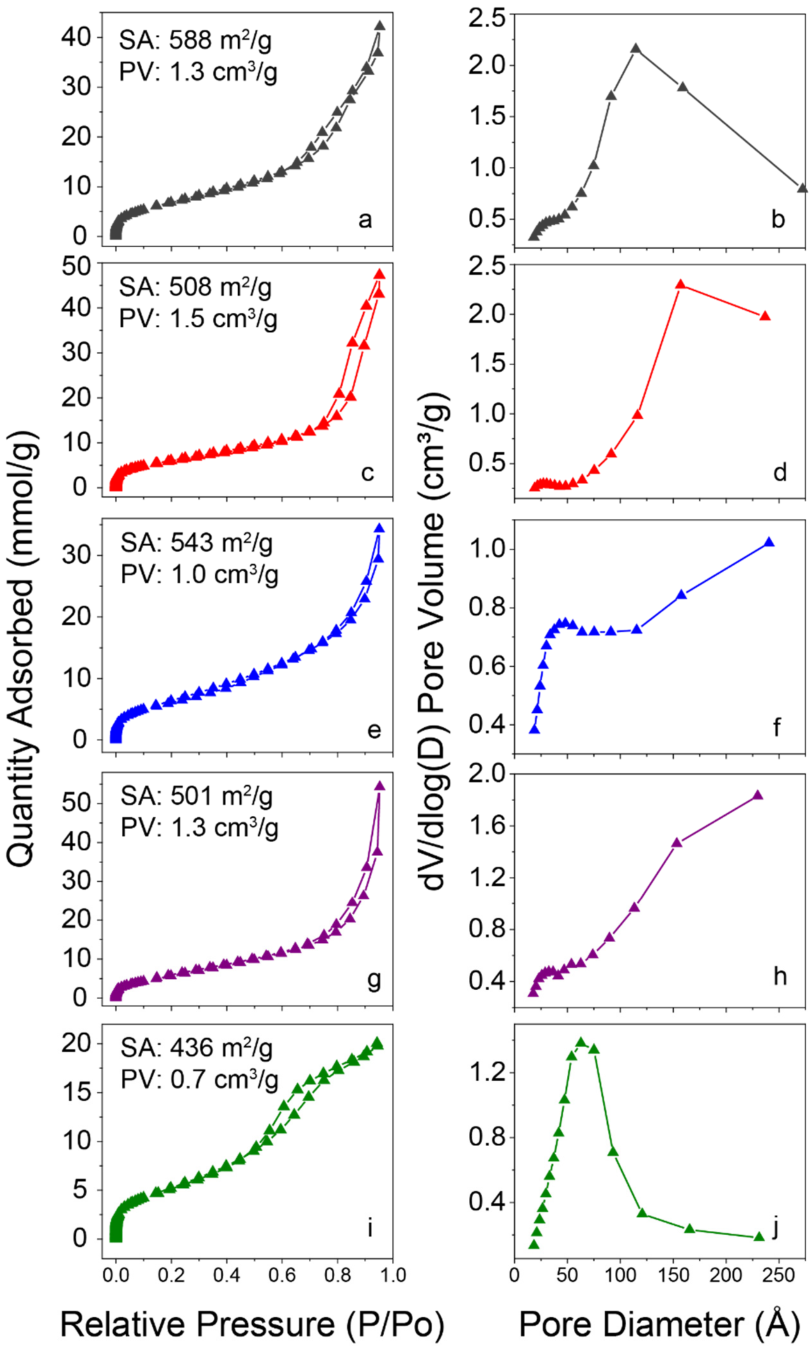

Supplementary Figure 4. N_2 sorption isotherm (1st column) and BJH adsorption pore size distribution (2nd column) of the synthesized AAS, (a,b) AC*-1.9, (c,d) AC-1.9, (e,f) AC-9, (g,h) AC-1.3, (i,j) IP-0.3.

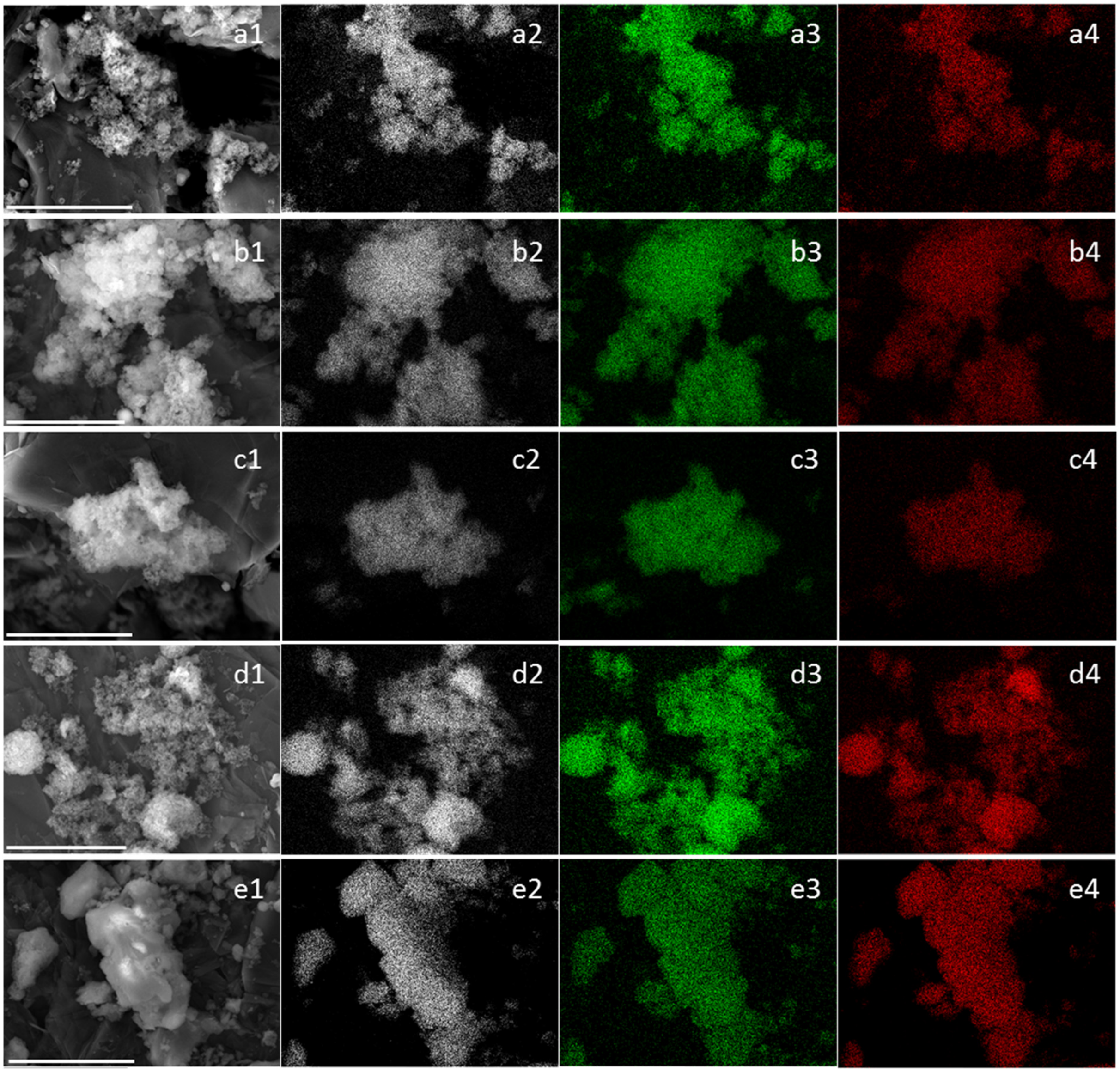

Supplementary Figure 5. SEM images with respective SEM-EDX elemental mapping of (a) AC*-1.9, (b) AC-1.9, (c) AC-9, (d) AC-1.3, (e) IP-0.3. In the EDX map, oxygen is represented as grey (a2-e2), silicon as green (a3-e3), and aluminum as red (a4-e4). Scale bar represents 5 μm.

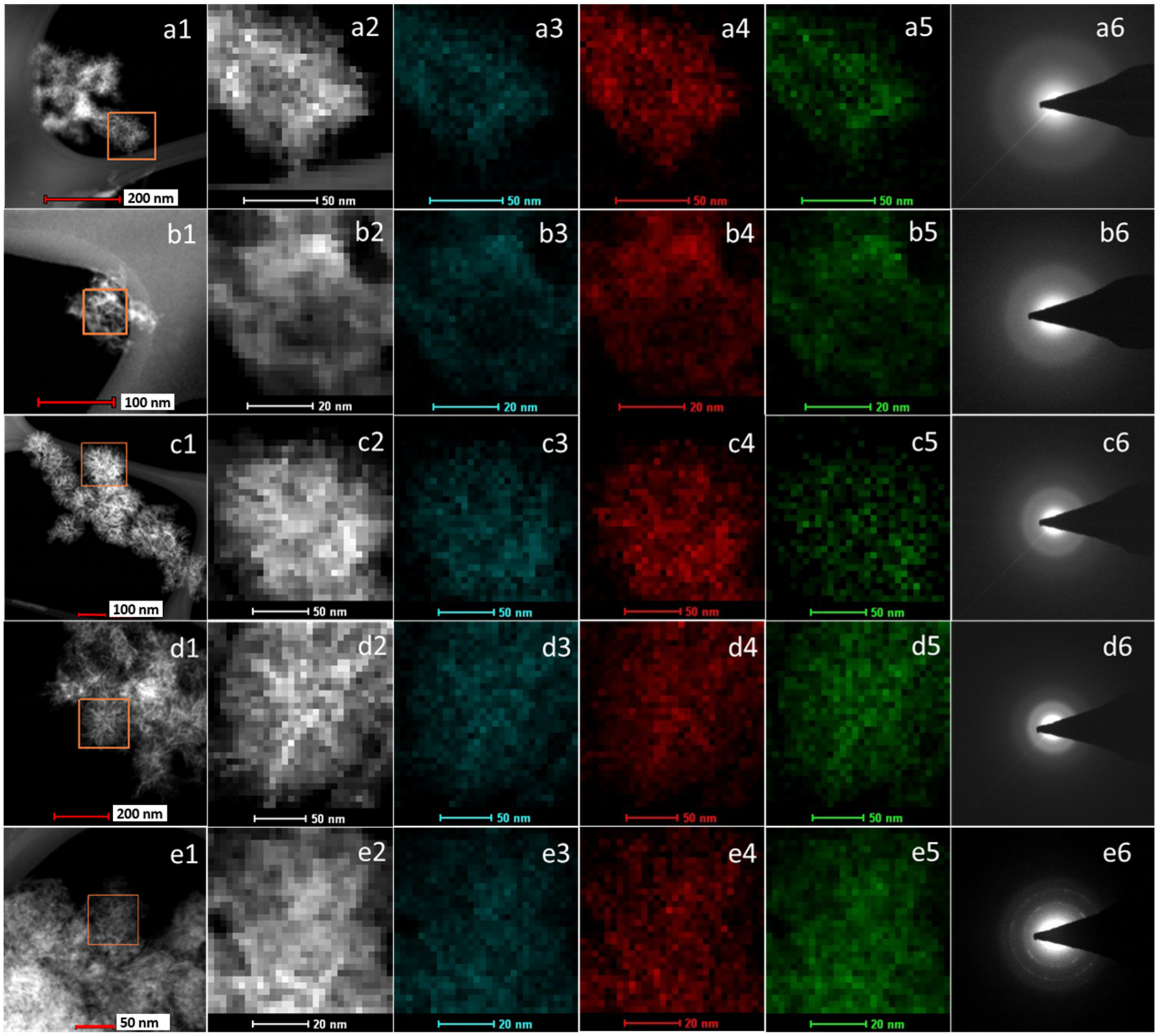

Supplementary Figure 6. TEM HAADF images (1 & 2 columns), TEM-EDX mapping (3-5 columns) and SAED (6 column) of (a) AC*-1.9, (b) AC-1.9, (c) AC-9, (d) AC-1.3, (e) IP-0.3. In the EDX map oxygen is represented as cyan (a3-e3), silicon as red (a4-e4) and aluminum as green (a5-e5).

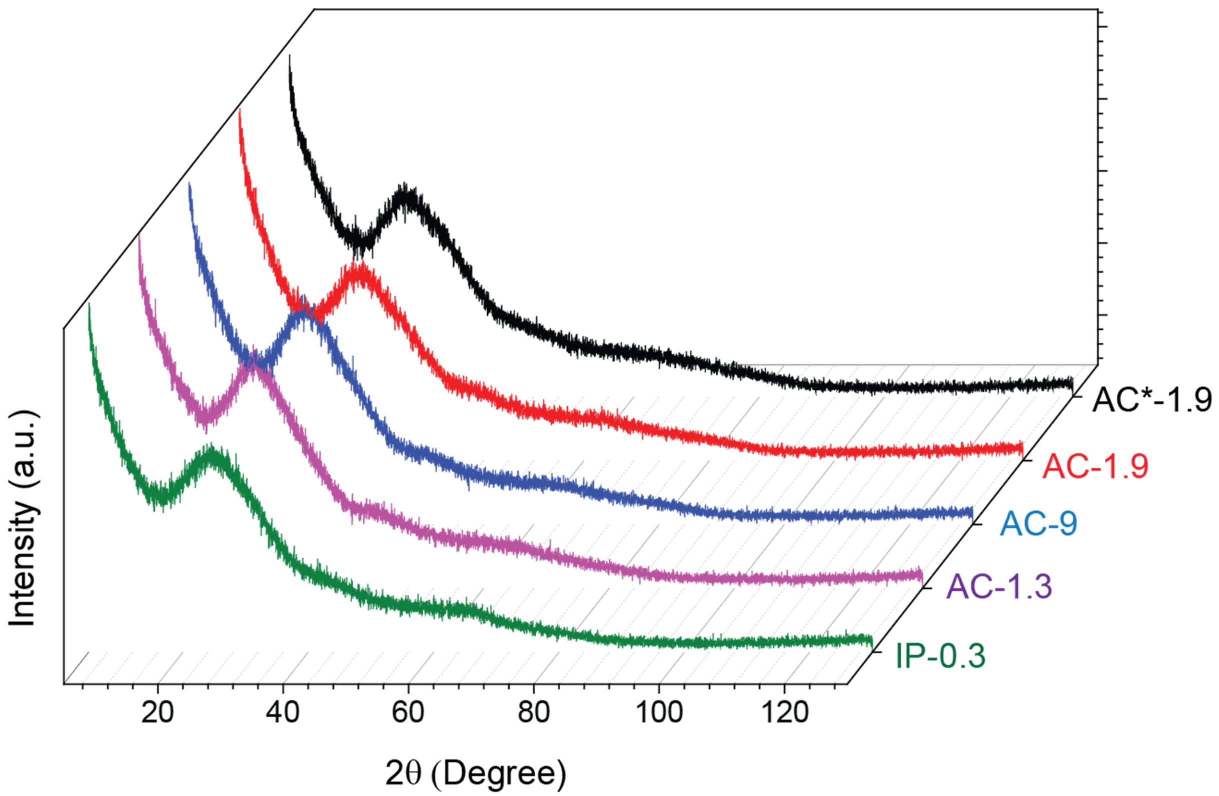

Supplementary Figure 7. Powder XRD pattern of the synthesized AAS.

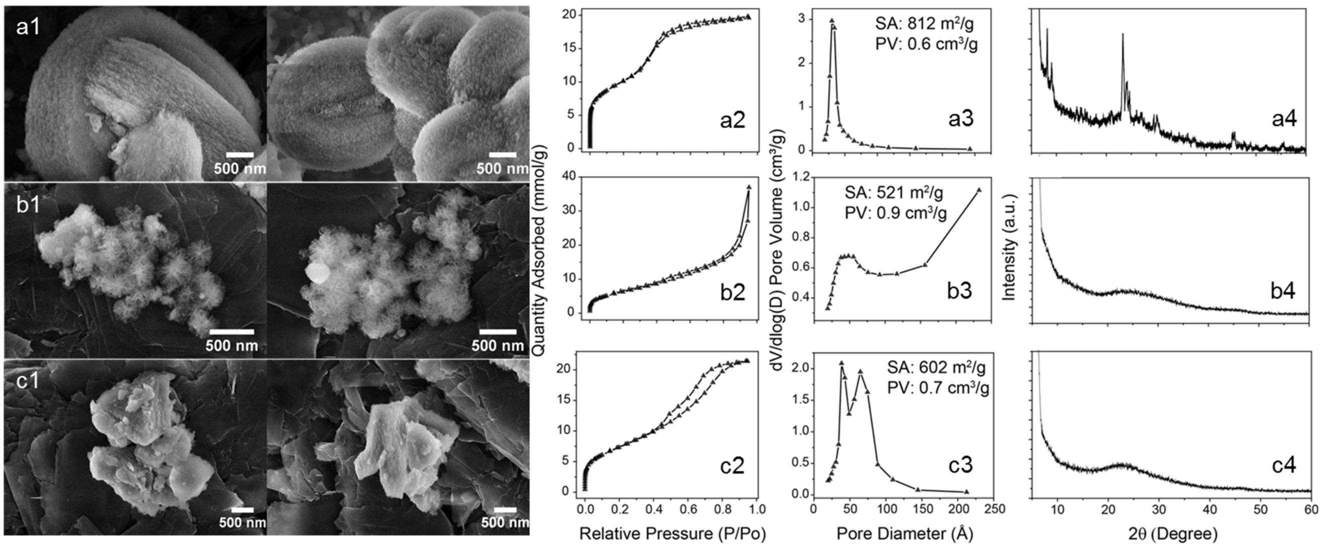

Supplementary Figure 8. SEM images (1-2 columns), N_2 adsorption isotherms (3rd column), BJH adsorption pore size distribution (4th column) and PXRD (5th column) of a) MFI-Meso-Zeolite, b) IP-2.8 and c) ASA-2.

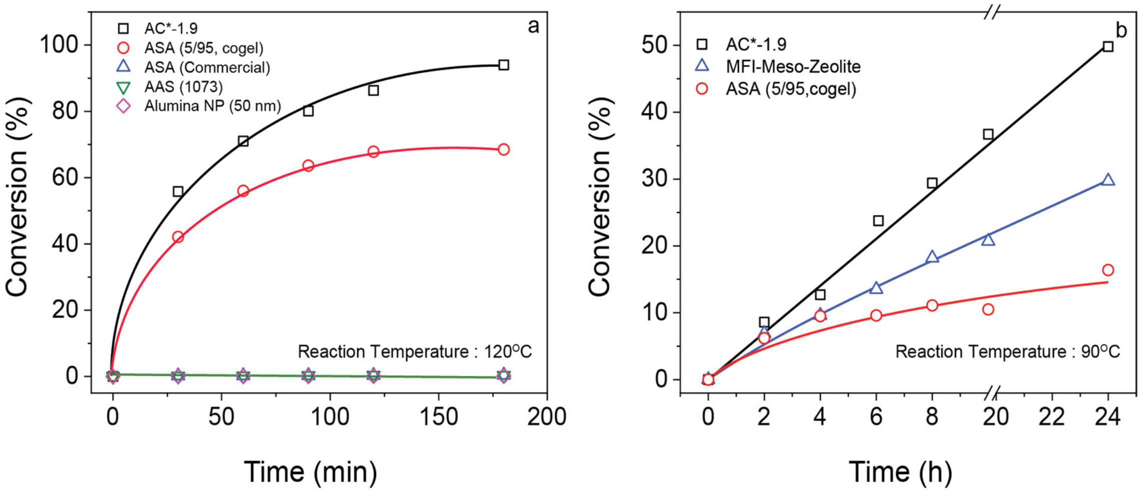

Supplementary Figure 9. Solid acid-catalyzed Friedel−Crafts alkylation of anisole by benzyl alcohol, a) reaction kinetics at 120 °C, b) reaction kinetics at 90°C. Error in the conversion was ± 10 %.

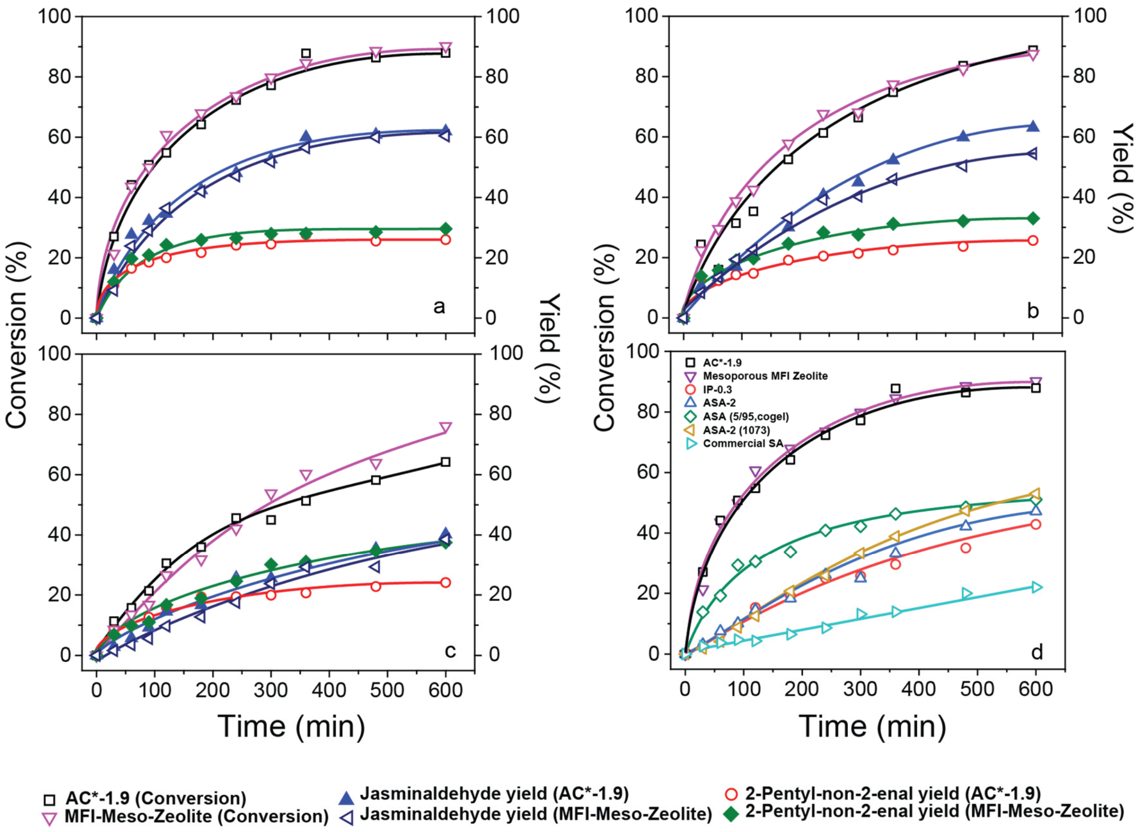

Supplementary Figure 10. Jasminaldehyde synthesis using solid acids, conversion and yield of the products at 125°C (a) after activation, (b) without activation; (c) conversion and yield of the products at 100°C after activation; (d) comparison with reported solid acids at 125°C after activation.

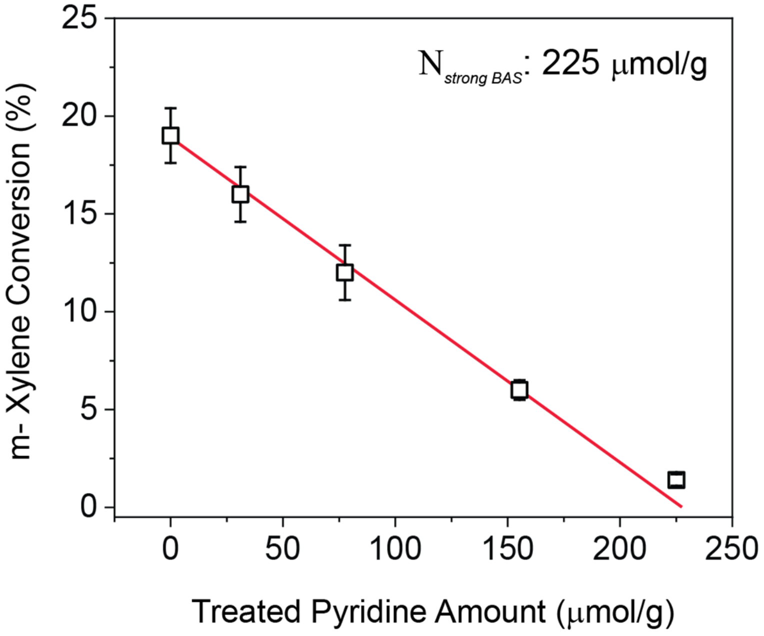

Supplementary Figure 11. m-Xylene isomerization by AC*-1.9 after treatment with varying amounts of pyridine.

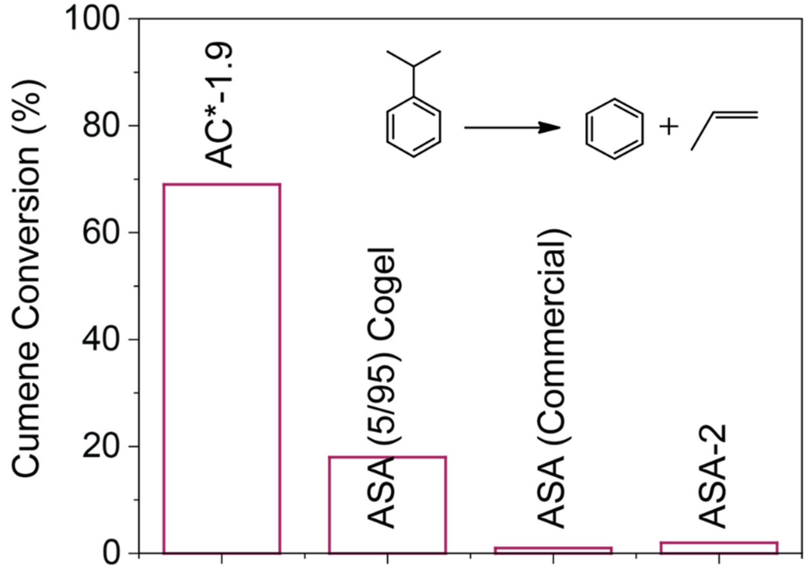

Supplementary Figure 12. Cumene cracking by various amorphous aluminosilicates at 300°C.

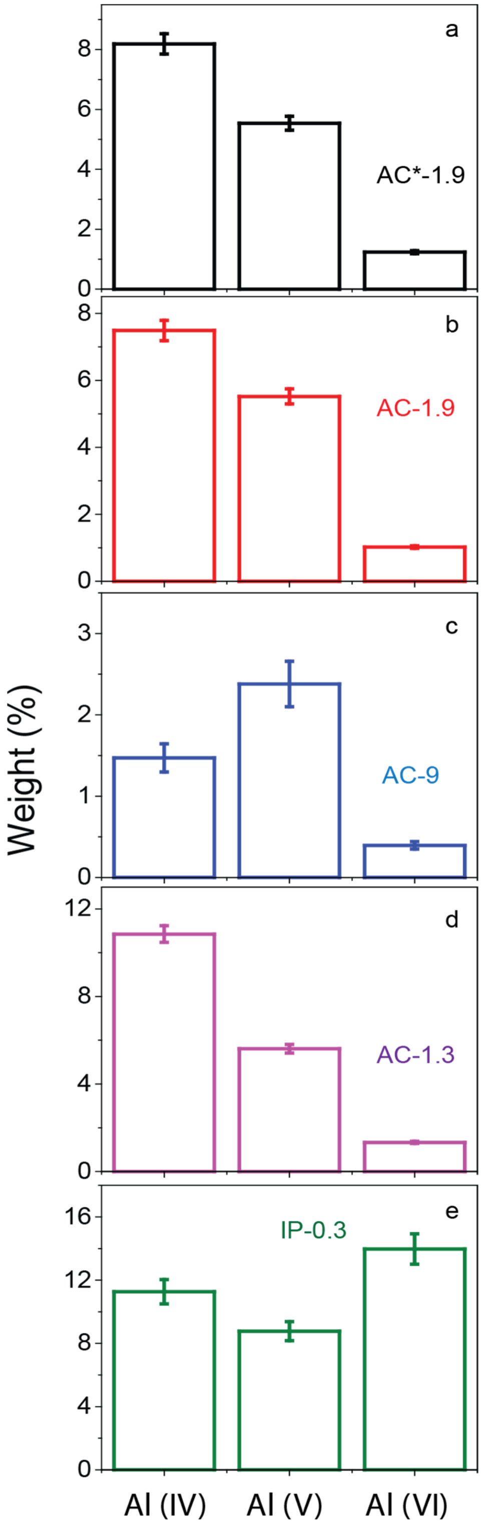

Supplementary Figure 13. Weight (%) of individual Al sites obtained from EDX and 1D single-pulse Al-MAS spectra, (a) AC*-1.9, (b) AC-1.9, (c) AC-9, (d) AC-1.3, (e) IP-0.3.

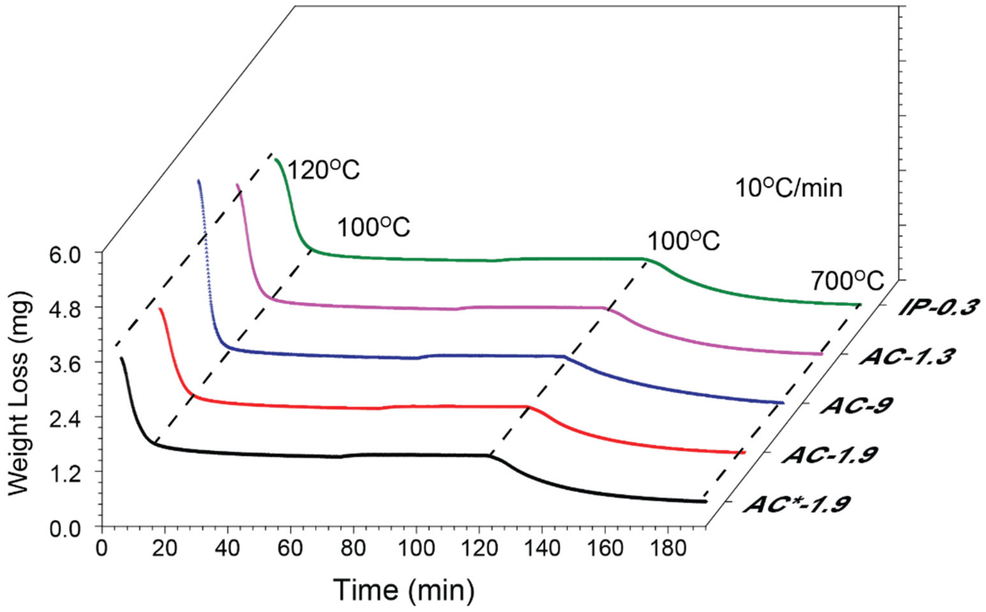

Supplementary Figure 14. Thermogravimetric analysis of various AAS.

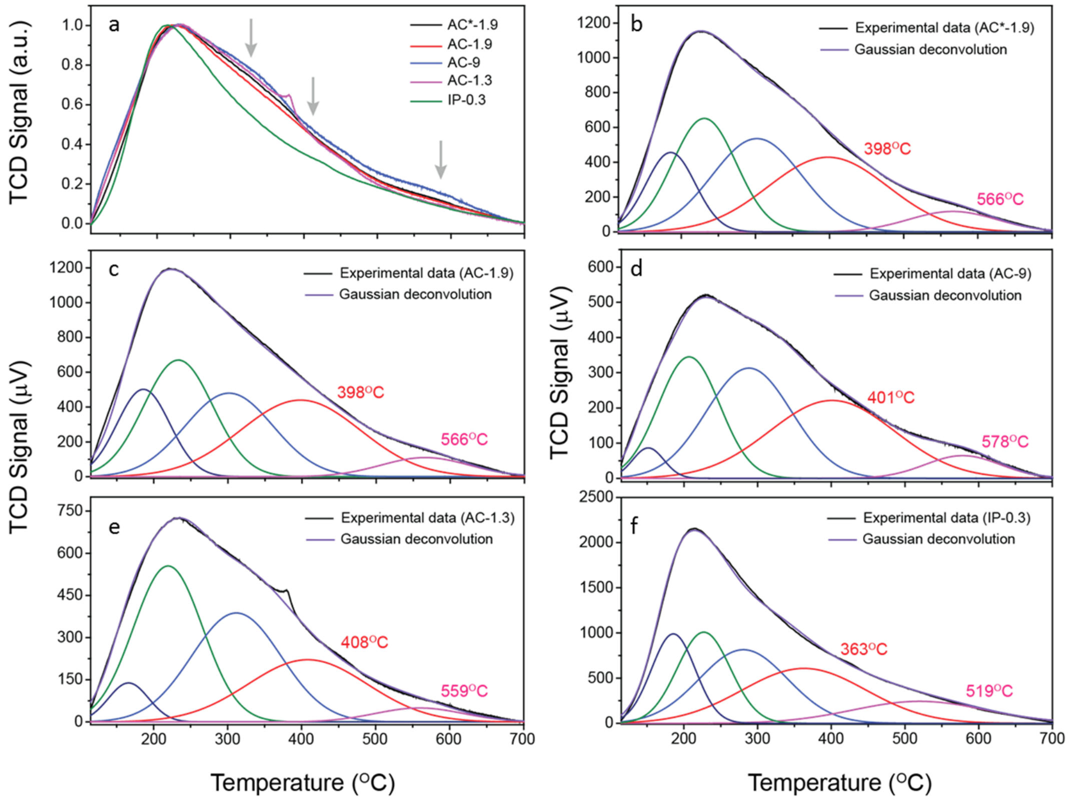

Supplementary Figure 15. Ammonia TPD (a) overlapped traces of the various AAS, and traces of individual AAS (b) AC*-1.9, (c) AC-1.9, (d) AC-9, (e) AC-1.3, and (f) IP-0.3

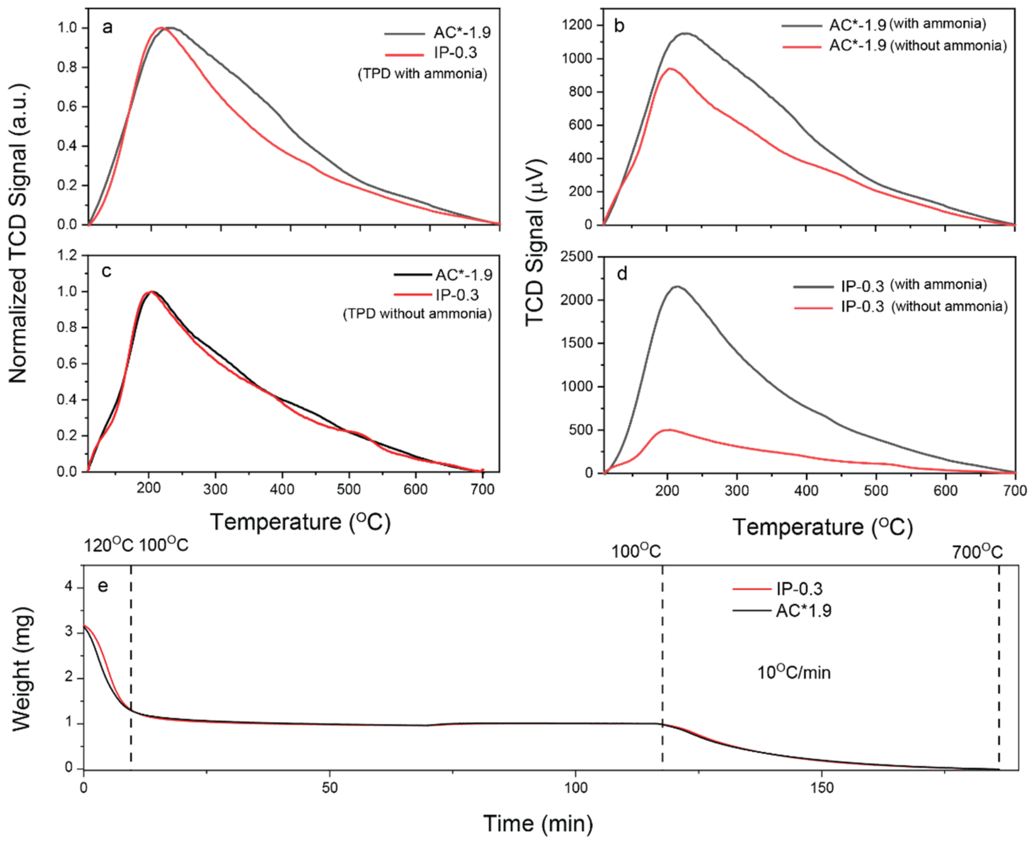

Supplementary Figure 16. a) Ammonia TPD traces of AC*-1.9 and IP-0.3, b) Overlapped TPD traces of AC*-1.9 with ammonia and without ammonia, c) TPD traces of AC*-1.9 and IP-0.3 without ammonia d) Overlapped TPD traces of IP-0.3 with ammonia and without ammonia, e) TGA profile of AC*-1.9, and IP-0.3 under the exact conditions that of TPD measurements.

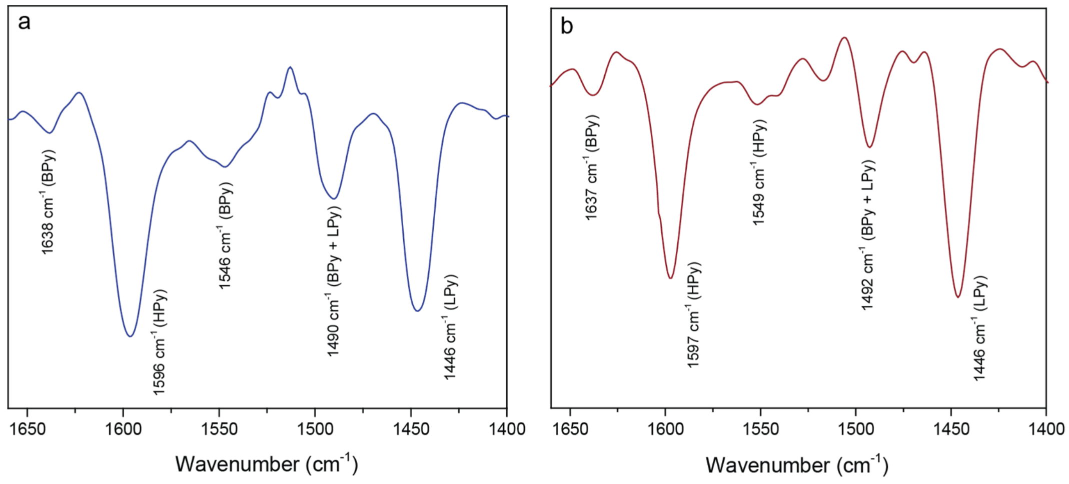

Supplementary Figure 17. DRIFT spectra of adsorbed pyridine on AC*-1.9, after a) pyridine desorption at 393K and b) pyridine desorption performed at 523K.

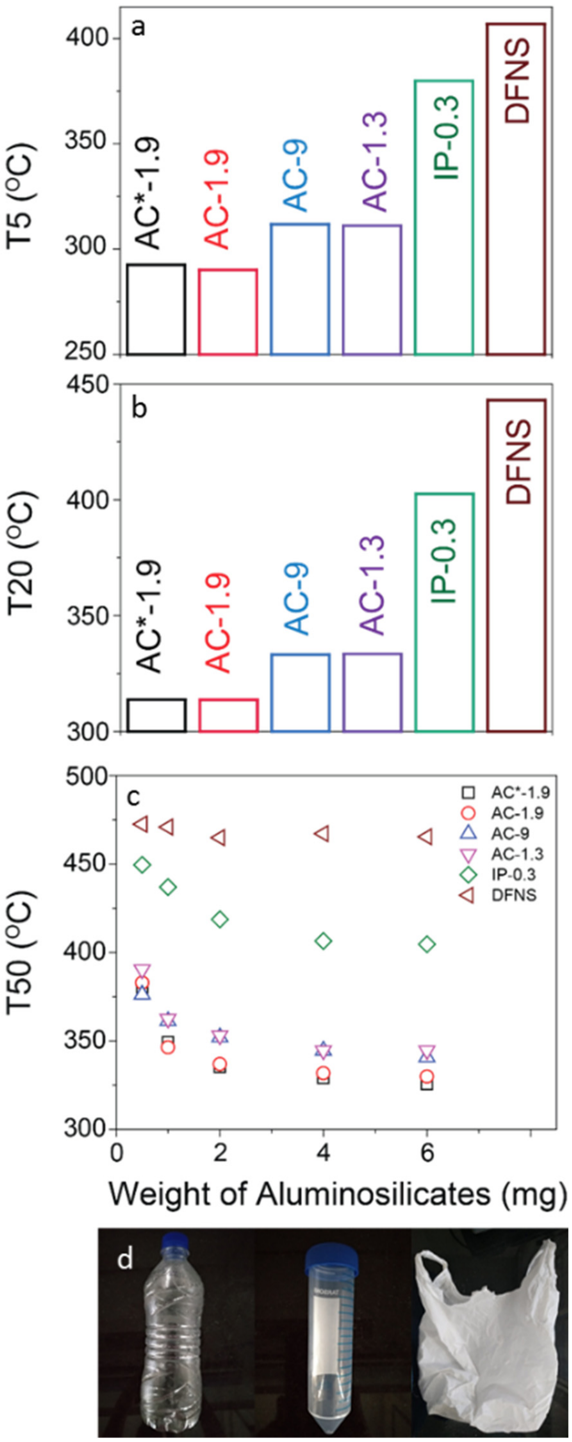

Supplementary Figure 18. LDPE (6 mg) pyrolysis using AAS (2 mg), (a) T5, (b) T20, (c) T50 with varying AAS amount, (d) photograph of the plastic bottle, centrifuge tube (falcon tube), carry bag used for the pyrolysis experiment.

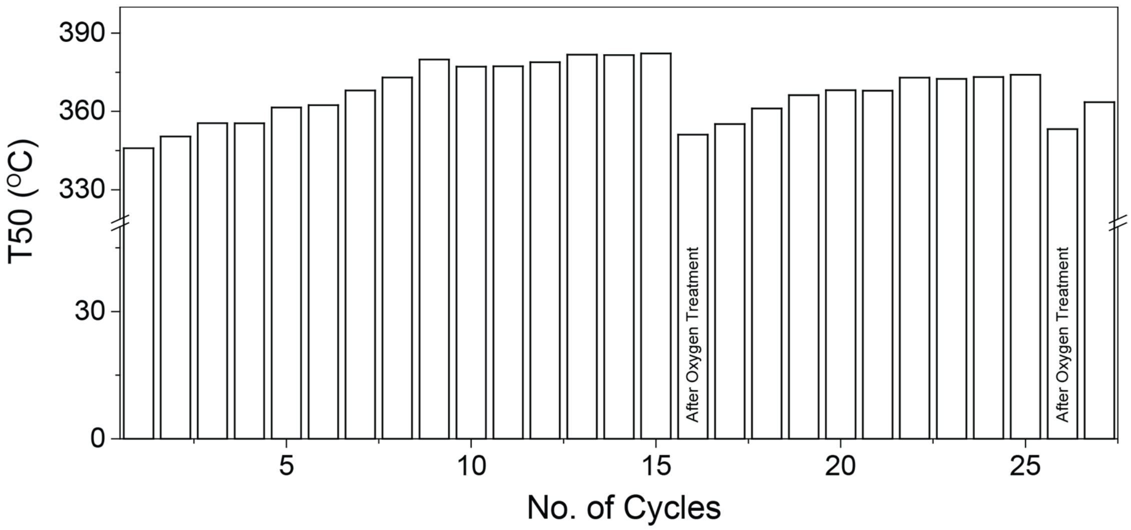

Supplementary Figure 19. Recyclability study of AC*-1.9 for LDPE degradation. Heated in oxygen (40 mL min^{-1}) from 30 to 800 °C (ramp rate 10°C min^{-1}) to regenerate the catalyst.

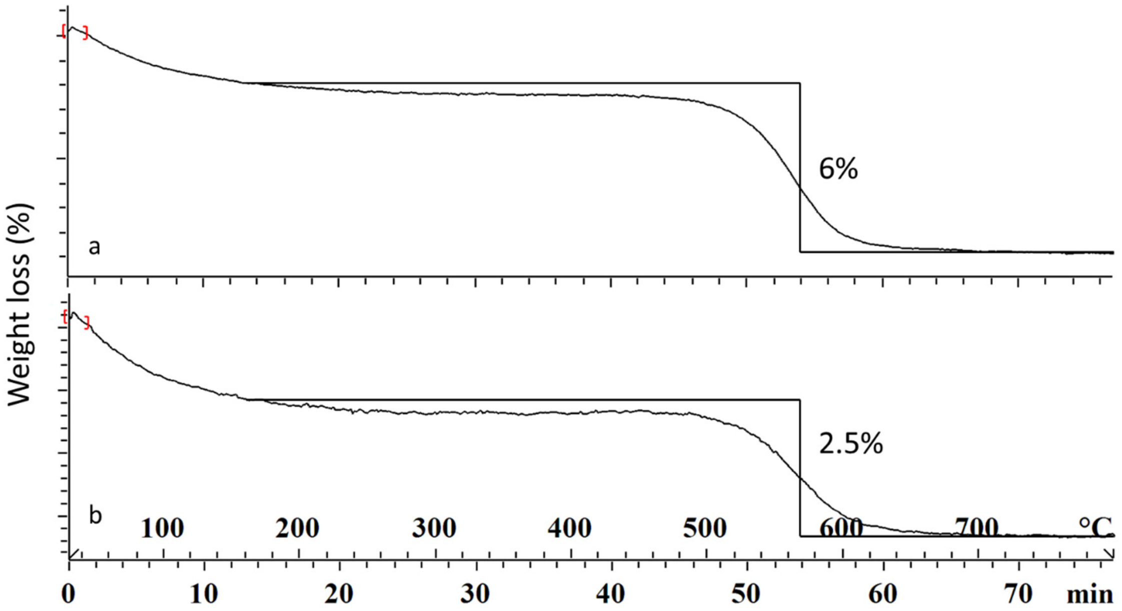

Supplementary Figure 20. Thermal gravimetric analysis (TGA) of the AC*-1.9 after (a) 15th cycle and (b) 25th cycle of LDPE pyrolysis.

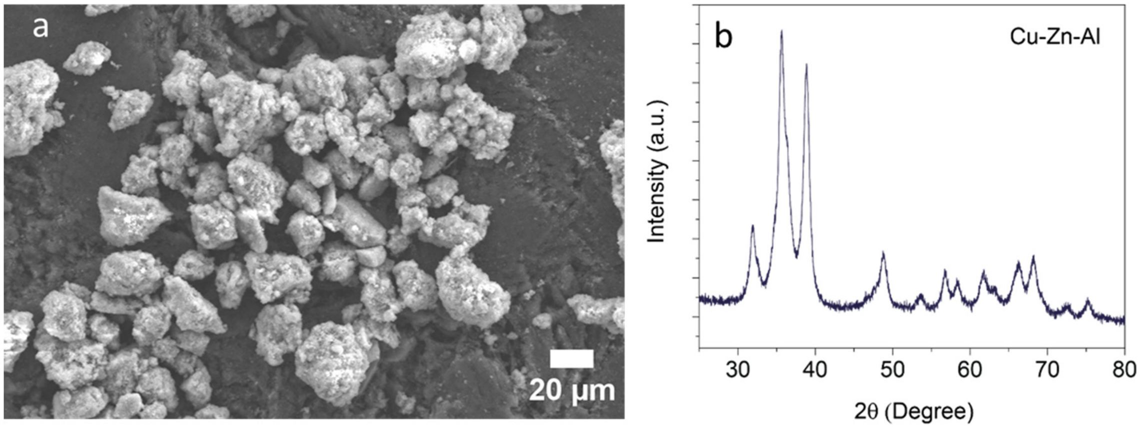

Supplementary Figure 21. (a) SEM and (b) PXRD of Cu-Zn-Al

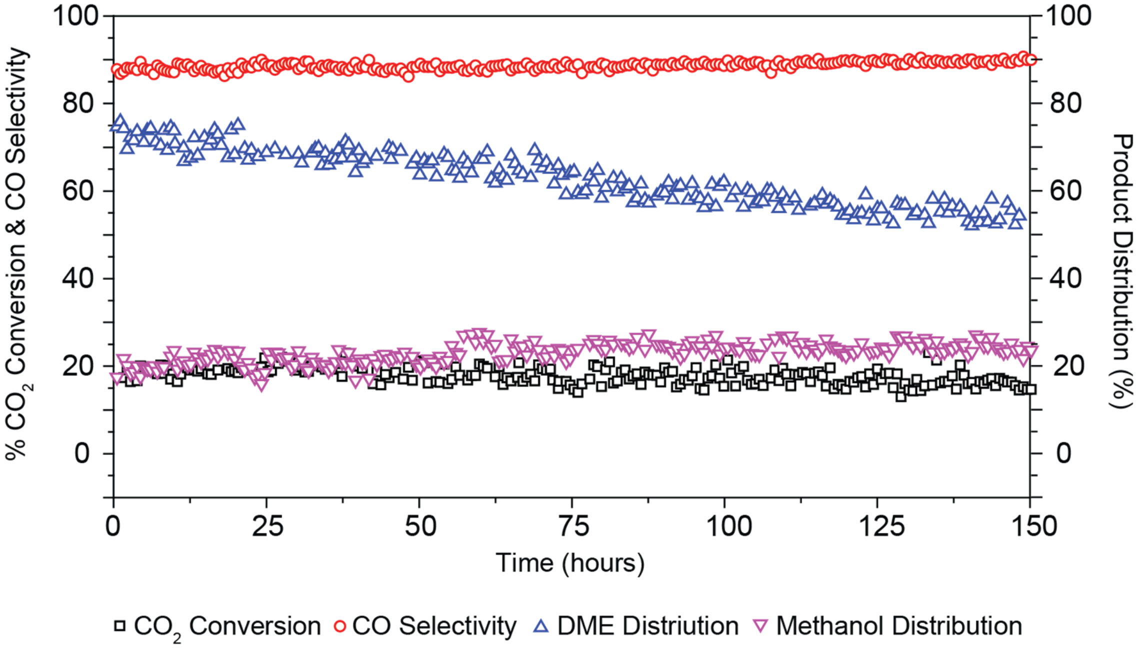

Supplementary Figure 22. Stability study of the catalyst (Cu-Zn-Al/AC*-1.9) at GHSV of 1500 mL $h^{-1}g^{-1}$ for 150 h under the stream.

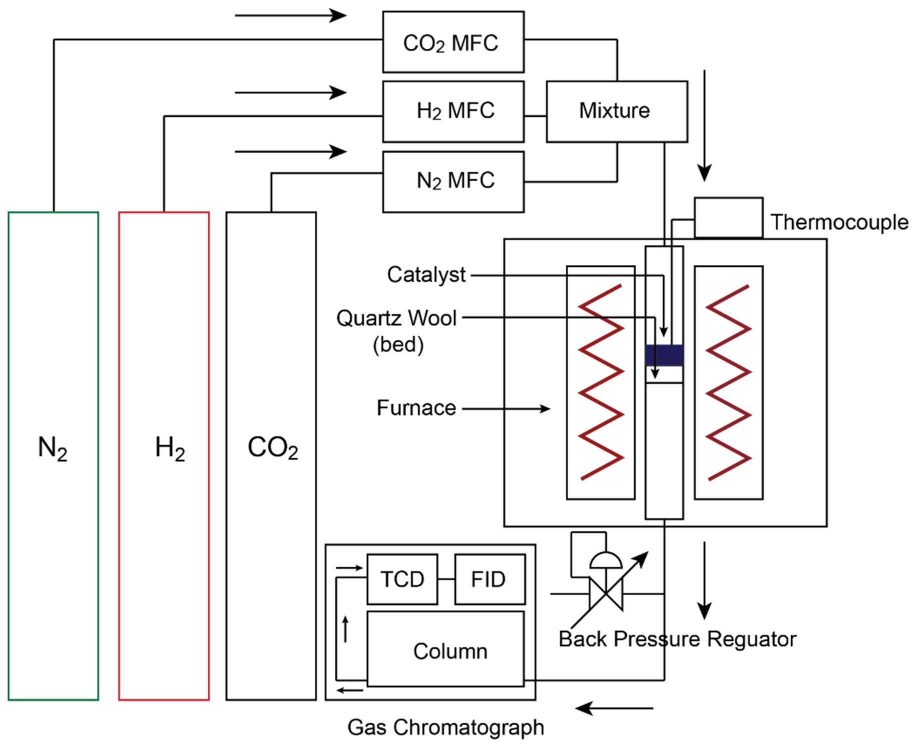

Supplementary Figure 23. Schematic design of the flow reactor used for CO_2 to DME conversion using hybrid bi-functional catalysts Cu-Zn-Al/AAS.

Supplementary Tables

Supplementary Table 1. Elemental analysis by EDX of the synthesized AAS

Catalyst	Si (wt%)	Al (wt%)	O (wt%)	Si/Al (atomic)	SiO_2/Al_2O_3 (weight)
AC*-1.9	29.2 ±1.3	15.0 ±0.6	55.8 ±1.6	1.9	2.2
AC-1.9	28.3 ±0.9	14 ±0.6	57.7 ±1.8	1.9	2.2
AC-9	39.0 ±2.7	4 ±0.5	57 ±2.8	9	10.2
AC-1.3	25 ±1	18 ±0.6	57 ±1	1.3	1.5
IP-0.3	11.2 ±1.3	34 ±2.3	54.8 ±2.0	0.3	0.35
MFI-Meso-Zeolite	55±1.5	3±0.5	42±1.5	14	16
IP-2.8	31±0.9	11±0.6	58±1.5	2.8	3.2
ASA-2	28±0.8	14±0.5	58±1.8	2	2.3

Supplementary Table 2. Friedel–Crafts alkylation of anisole by benzyl alcohol using various AAS under microwave irradiation

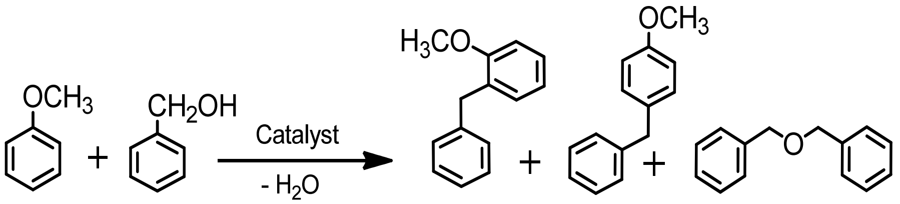

Catalyst	Temperature (°C)	Time (min)	Amount (mg)	Conversion (%)
AC*1.9	160	15	25	100
AC-1.9	160	15	25	100
AC-9	160	15	25	100
AC-1.3	160	15	25	100
IP-0.3	160	15	25	30

Supplementary Table 3. Friedel−Crafts alkylation of anisole by benzyl alcohol using various AAS in an oil bath at 120 °C

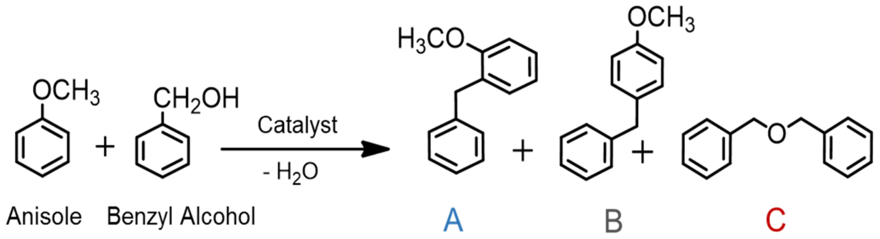

Entry No.	Catalyst	Conversion (%)	Selectivity (%)		
			A	B	C
1	AC*-1.9	94	48	47	5
2	AC-1.9	57	40	45	15
3	AC-9	80	40	45	15
4	AC*-1.3	34	41	47	12
5	Meso-MFI-Zeolite	68	44	46	10
6	ZSM-5	2	30	49	21
7	IP-0.3	2	34	20	46
8	IP-2.8	7	40	48	12
9	ASA-2	2	46	26	27

Supplementary Table 4. OH density estimation of various AAS catalysts from TGA

Catalyst	OH/nm^2
AC*-1.9	7.6
AC-1.9	9.5
AC-9	5.4
AC-1.3	7.9
IP-0.3	13

Supplementary Table 5. Peak area of various peaks of the AAS TPD spectra

Sample Name	Peak 1: Area (Temp °C)	Peak 2: Area (Temp °C)	Peak 3: Area (Temp °C)	Peak 4: Area (Temp °C)	Peak 5: Area (Temp °C)
AC*-1.9	38098 (186)	70550 (231)	81300 (302)	87064 (398)	17547 (566)
AC-1.9	44886 (186)	78451 (233)	71532 (302)	89314 (398)	16392 (566)
AC-9	4596 (151)	35993 (207)	45425 (288)	47034 (401)	8241 (578)
AC-1.3	9452 (166)	64964 (217)	58592 (312)	44774.6 (408)	7425 (559)
IP-0.3	73688 (186)	909223 (227)	121826 (281)	127801 (363)	55152 (519)

Supplementary Table 6. Chemical composition of the plastic used for this degradation studies

Plastic Bottle (Polyethylene Terephthalate,PET)	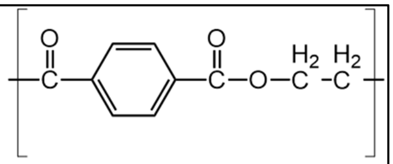
Centrifuge tube (Polypropylene)	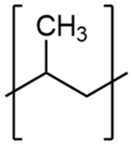
Carry bag (High density polyethylene)	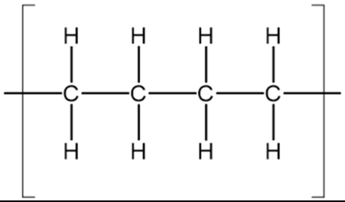

Supplementary Table 7. T50 comparison of different solid acids for LDPE degradation

Catalyst	T50 (°C)	LDPE (mg)	Aluminosilicate (mg)
AC*-1.9	335	6	2
AC*-1.9	325	6	6
AC*-1.9	350	6	1
AC*-1.9	380	6	0.5
AC-1.9	337	6	2
AC-9	352	6	2
AC-1.3	353	6	2
IP-0.3	420	6	2

IP-2.8	410	6	2
DFNS	465	6	2
USY-40[50]	341	6	2
USY-2.6[50]	377	6	2
ASA (commercial) [50]	360	6	2
S-0.03-NaOH[50]	336	6	2
Meso-MFI-Zeolite	354	6	2
ZSM-5	424	6	2
ASA-2	417	6	2

Ref 50. Locus, R. et al. Synthetic and catalytic potential of amorphous mesoporous aluminosilicates prepared by postsynthetic aluminations of silica in aqueous media. ChemCatChem 10, 1385–1397 (2018).

Supplementary Table 8. Elemental composition of Cu-Zn-Al catalyst by EDX

Sample Name	Cu	Zn	Al	O
Cu-Zn-Al (atomic %)	25.7	13.7	4.4	56.2
Cu-Zn-Al (weight %)	45.8	25.3	3.3	25.6
